# Supplementary material for: Nur77 suppresses hepatocellular carcinoma via switching glucose metabolism toward gluconeogenesis through attenuating phosphoenolpyruvate carboxykinase sumoylation
Source: Nat Commun. 2017 Feb 27;8:14420. doi: 10.1038/ncomms14420 (PMC5333363; doi:10.1038/ncomms14420)
Supplement: Supplementary Information — Supplementary Figures and Supplementary Tables [file ncomms14420-s1.pdf]

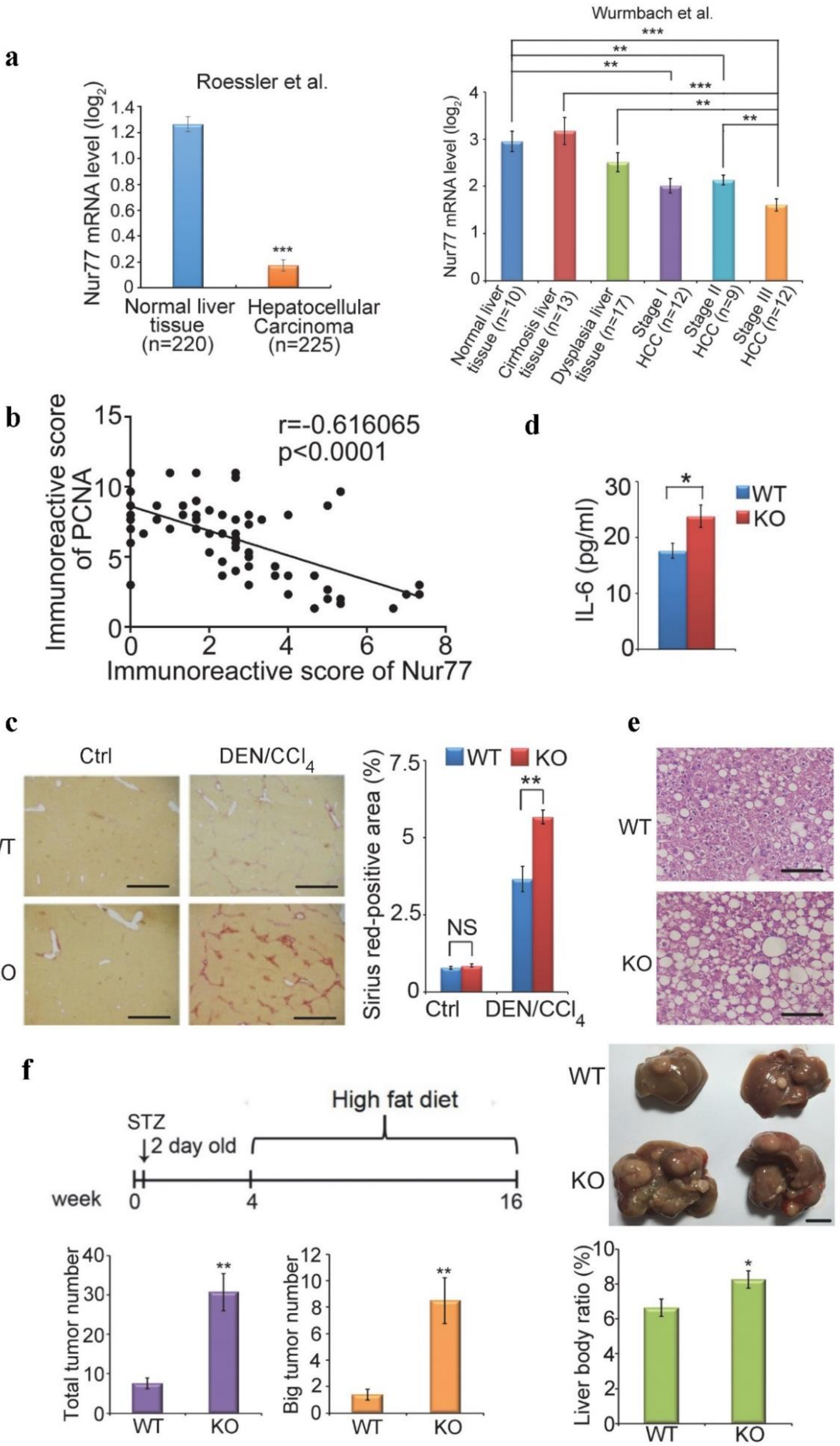
a
Roessler et al.
Nur77 mRNA level (log2)
Normal liver tissue (n=220)
Hepatocellular Carcinoma (n=225)
Wurmbach et al.
Normal liver tissue (n=10)
Cirrhosis liver tissue (n=13)
Dysplasia liver tissue (n=17)
Stage I HCC (n=12)
Stage II HCC (n=9)
Stage III HCC (n=12)
b
r=-0.616065
p<0.0001
Immunoreactive score of PCNA
Immunoreactive score of Nur77
d
IL-6 (pg/ml)
WT
KO
c
Ctrl
DEN/CCl4
WT
KO
Sirius red-positive area (%)
NS
e
WT
KO
f
High fat diet
STZ
2 day old
week
0
4
16
Total tumor number
Big tumor number
Liver body ratio (%)
WT
KO

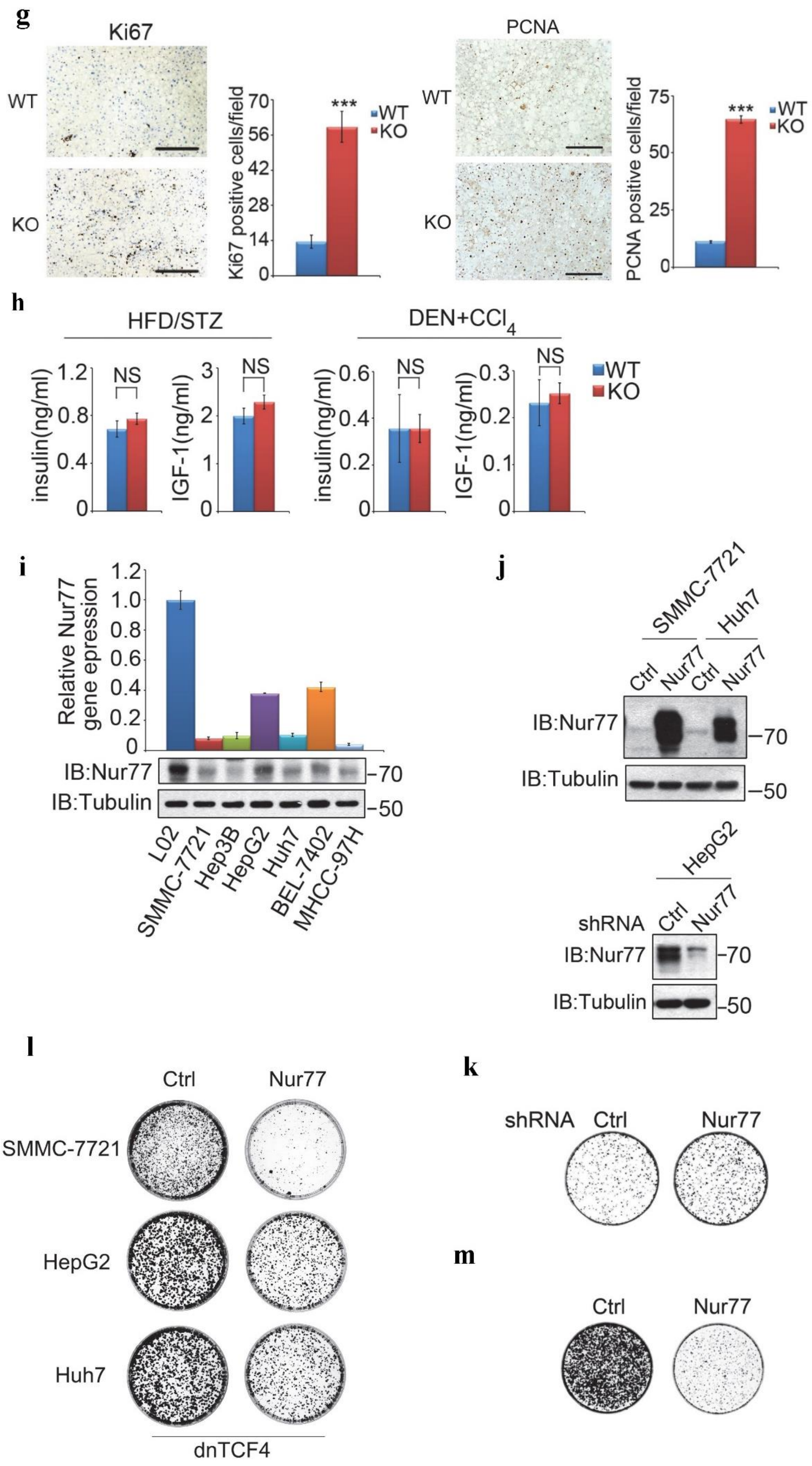
g
Ki67
WT
KO
Ki67 positive cells/field
0
14
28
42
56
70
***
WT
KO
PCNA
WT
KO
PCNA positive cells/field
0
25
50
75
***
WT
KO
h
HFD/STZ
DEN+CCl4
insulin(ng/ml)
0
0.4
0.8
1.2
NS
IGF-1(ng/ml)
0
1
2
3
NS
insulin(ng/ml)
0
0.2
0.4
0.6
NS
IGF-1(ng/ml)
0
0.1
0.2
0.3
NS
WT
KO
i
Relative Nur77 gene epression
0
0.2
0.4
0.6
0.8
1.0
1.2
IB:Nur77
–70
IB:Tubulin
–50
L02
SMMC-7721
Hep3B
HepG2
Huh7
BEL-7402
MHCC-97H
j
SMMC-7721
Huh7
Ctrl
Nur77
Ctrl
Nur77
IB:Nur77
–70
IB:Tubulin
–50
HepG2
shRNA
Ctrl
Nur77
IB:Nur77
–70
IB:Tubulin
–50
l
Ctrl
Nur77
SMMC-7721
HepG2
Huh7
dnTCF4
k
shRNA
Ctrl
Nur77
m
Ctrl
Nur77

Supplementary Figure 1.

(a) The levels of Nur77 mRNA in hepatocellular carcinoma and normal liver (left), and different stages of tumor development (right). The data are obtained from Roessler Liver 2 dataset and Wurmbach Liver dataset of Oncomine database, respectively.

(b) The negative correlation of protein expression levels between Nur77 and PCNA in HCC samples (n=66).

(c) Comparison of hepatic fibrosis in DEN/CCl_4-induced HCC samples between WT and Nur77 KO mice at 12-week-old, detected by Picro Sirius Red staining. The Sirius Red positive areas were quantified in 20 randomly selected fields/per mouse (n=6). Scale bars, 1 mm.

(d) Comparison of serum IL-6 levels in DEN/CCl_4-induced HCC model between WT (n=7) and Nur77 KO (n=10) mice at 22-week-old, detected by ELISA.

(e) Comparison of hepatic steatosis in HFD/STZ model between WT and Nur77 KO mice at 12-week-old were detected by H&E staining of liver tissues. Scale bars, 50 μm.

(f) Top, the schematic overview of HFD/STZ HCC mice model (left) and liver images (right). Bottom, total tumor number, big tumor (diameter > 5 mm) numbers and liver:body weight ratio are demonstrated (n=10). Pups from WT and Nur77 KO male mice were injected with streptozotocin (STZ, 200 μg/per mouse) at the age of 2 days, and followed by 12 weeks of high-fat diet from the age of 4 weeks. Scale bars, 1 cm

(g) Ki67 and PCNA expression levels are indicated in HFD/STZ-induced HCC samples from WT and Nur77 KO mice, and positive cells were quantified in 20 randomly selected fields/per mouse (n=6). Scale bars, 100 μm

(h) Comparison of serum insulin and IGF1 levels between WT and Nur77 KO mice of DEN/CCl_4- or HFD/STZ-induced HCC model (n=6).

(i) Analysis of Nur77 mRNA and protein levels in different cell lines. Quantitative real-time PCR and western blot were performed to determine Nur77 mRNA and protein levels.

(j) Stable overexpression of Nur77 (top) or knock-down of endogenous Nur77 by shRNA (bottom) are established in different cell lines. Nur77 expression levels were determined by western blot.

(k) Effect of Nur77 on proliferation of normal liver cells L02, determined by colony formation.

(l) Wnt signaling was inhibited by stable transfection of dnTCF4 in several HCC cells, and effect of Nur77 on cell proliferation was determined by colony formation assay.

(m) Effect of Nur77 on proliferation of p53-null Hep3B HCC cells was determined by colony formation assay.

Tubulin was used to indicate the amount of loading proteins. Data were represented as means ± SEM of at least three independent experiments. *: $p < 0.05$; **: $p < 0.01$; ***: $p < 0.001$. The data were analyzed using two-tailed Student’s t-test in **a** (left)**, d, f-h,** one-way ANOVA followed by Tukey post hoc test in **a** (right), **c** and Pearson's chi-squared test in **b**.

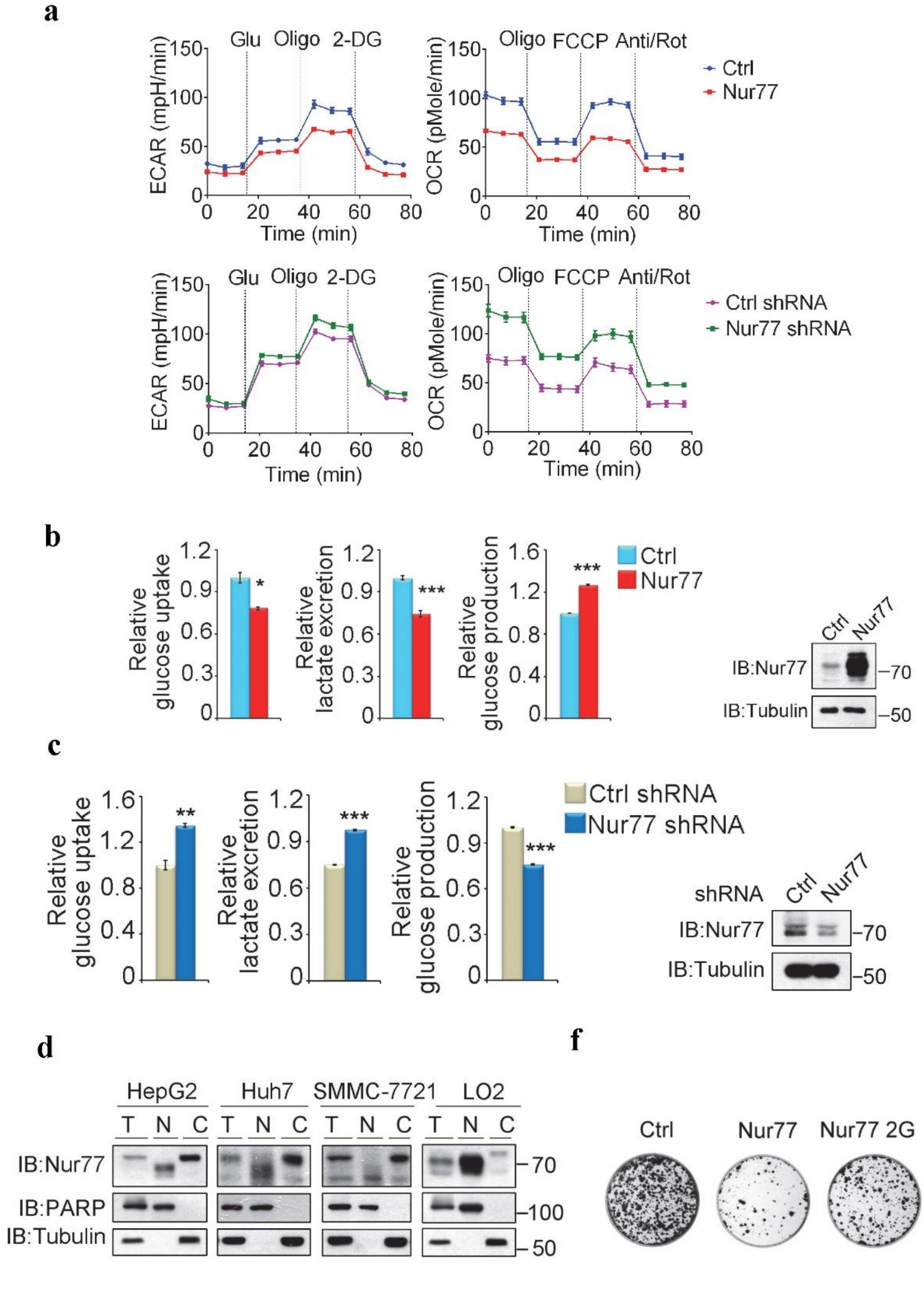
a
Glu Oligo 2-DG
ECAR (mpH/min)
Time (min)
Oligo FCCP Anti/Rot
OCR (pMole/min)
Ctrl
Nur77
Ctrl shRNA
Nur77 shRNA
b
Relative glucose uptake
Relative lactate excretion
Relative glucose production
Ctrl
Nur77
IB:Nur77
IB:Tubulin
c
Ctrl shRNA
Nur77 shRNA
shRNA
d
HepG2
Huh7
SMMC-7721
LO2
T N C
IB:Nur77
IB:PARP
IB:Tubulin
f
Ctrl
Nur77
Nur77 2G

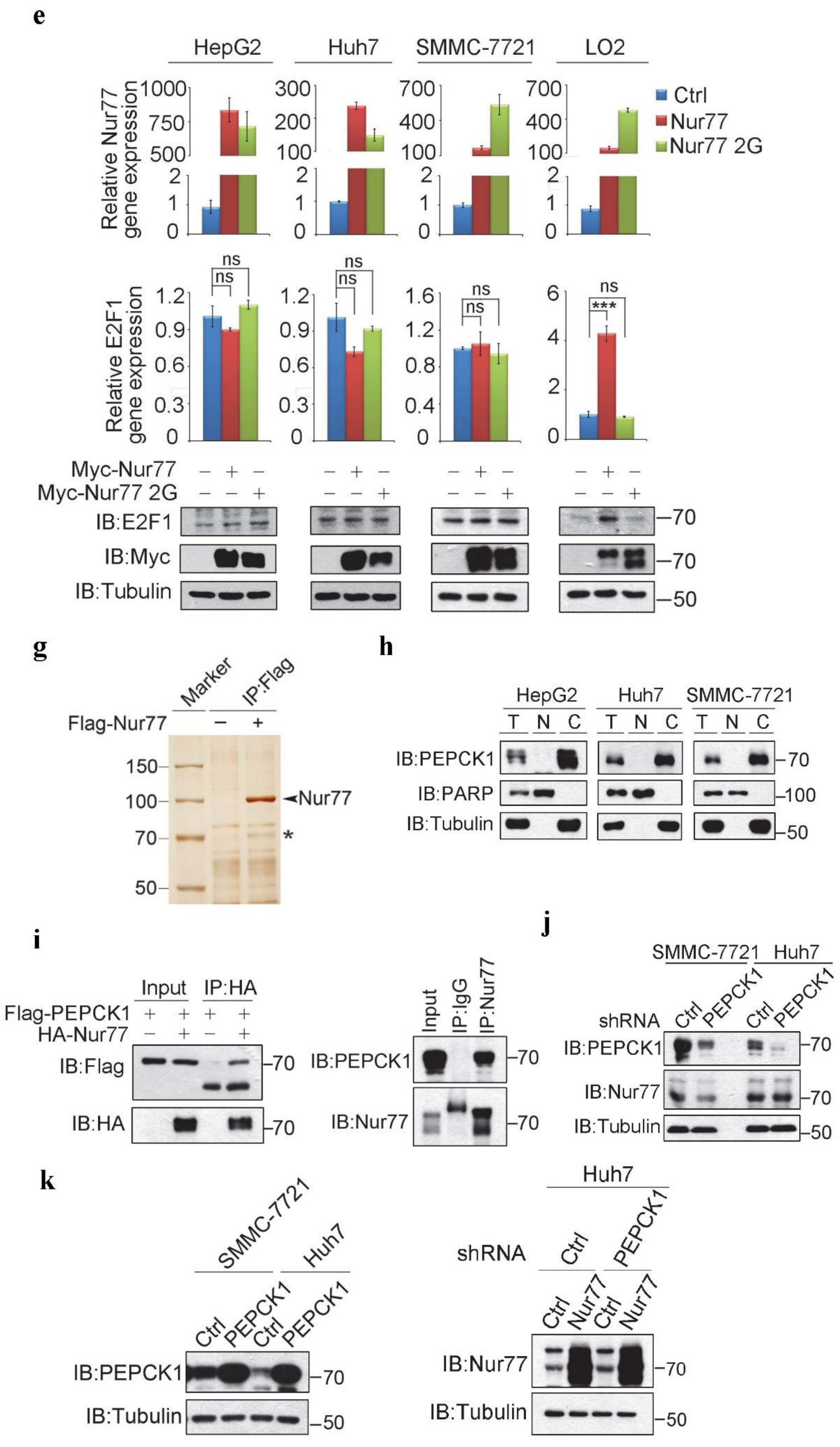
e
HepG2
Huh7
SMMC-7721
LO2
Relative Nur77 gene expression
Ctrl
Nur77
Nur77 2G
Relative E2F1 gene expression
ns
***
Myc-Nur77
Myc-Nur77 2G
IB:E2F1
IB:Myc
IB:Tubulin
g
Marker
IP:Flag
Flag-Nur77
Nur77
*
h
T N C
IB:PEPCK1
IB:PARP
i
Input
IP:HA
Flag-PEPCK1
HA-Nur77
IB:Flag
IB:HA
IP:IgG
IP:Nur77
IB:Nur77
j
shRNA
Ctrl
PEPCK1
k

l

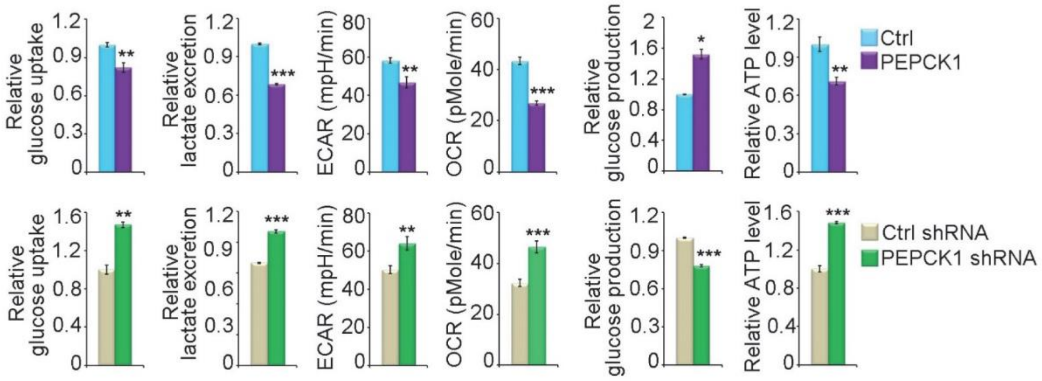
Relative glucose uptake
Relative lactate excretion
ECAR (mpH/min)
OCR (pMole/min)
Relative glucose production
Relative ATP level
Ctrl
PEPCK1
Ctrl shRNA
PEPCK1 shRNA

m

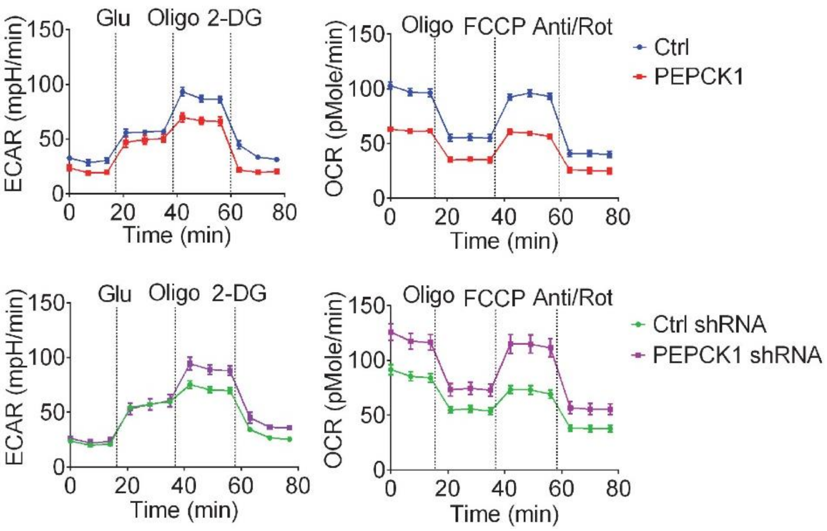
Glu Oligo 2-DG
Oligo FCCP Anti/Rot
ECAR (mpH/min)
OCR (pMole/min)
Time (min)
Ctrl
PEPCK1
Ctrl shRNA
PEPCK1 shRNA

n

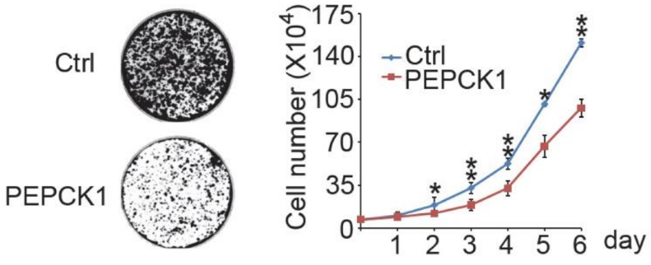
Ctrl
PEPCK1
Cell number (X10^4)
day

p

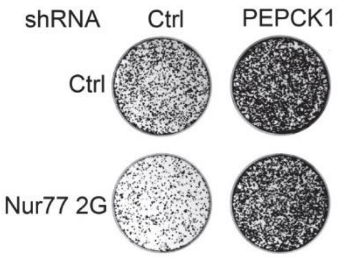
shRNA
Ctrl
PEPCK1
Ctrl
Nur77 2G

o

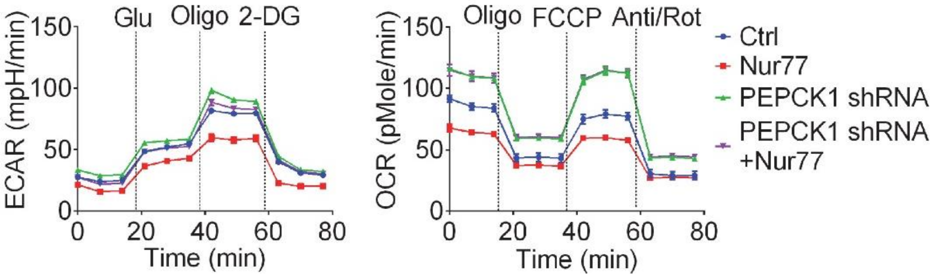
Glu Oligo 2-DG
Oligo FCCP Anti/Rot
ECAR (mpH/min)
OCR (pMole/min)
Time (min)
Ctrl
Nur77
PEPCK1 shRNA
PEPCK1 shRNA +Nur77

Supplementary Figure 2.

(a) The seahorse tracing curves for ECAR and OCR in Huh7 cells with Nur77 overexpression (top) or HepG2 cells with Nur77 knockdown (bottom).

(b) Detection of glucose uptake, lactate excretion, and glucose production in control and Nur77 overexpressing Hep3B cells those are deficient of p53.

(c) Detection of glucose uptake, lactate excretion, and glucose production in control and Nur77 knockdown normal liver L02 cells.

(d) Nur77 localized more in the cytoplasm in different HCC cell lines, but less in L02 cells. Nuclear and cytoplasmic fractions were prepared, and Nur77 localization was determined by western blot. PARP and Tubulin were used as the loading controls for nuclear (N), cytoplasmic (C) and total (T) cell extracts, respectively.

(e) Effects of Nur77 and Nur77 2G on expression levels of E2F1 gene (middle) and protein (bottom) in different liver cancer cell lines and normal liver L02 cell line. Expression levels of Nur77 and Nur77 2G were determined by real-time PCR (top).

(f) Nur77 2G inhibits SMMC-7721 cell proliferation determined by colony formation.

(g) Identification of PEPCK1 as a Nur77 binding partner. HEK293T cells were transfected with Flag-Nur77 or empty vector, and cell lysates were subjected for immunoprecipitation using anti-Flag antibody. Silver staining of Nur77-interacting complexes was shown. The specified band (indicated by an asterisk) was excised and analyzed by mass spectrometry.

(h) PEPCK1 exclusive localization in the cytoplasm in different HCC cell lines. Nuclear and cytoplasmic fractions were prepared, and PEPCK1 localization was determined by western blot. PARP and Tubulin were used as the loading controls for nuclear (N), cytoplasmic (C)

and total (T) cell extracts, respectively.

(i) Nur77 interacts with PEPCK1. Left, Flag-PEPCK1 and HA-Nur77 were transfected into 293T cells. Co-IP was performed to verify the interaction between PEPCK1 and Nur77. Right, endogenous Nur77 was immunoprecipitated, and then western blot was carried out to determine the *in vivo* Nur77-PEPCK1 interaction.

(j) Knocking-down PEPCK1 does not impair endogenous Nur77 expression level. PEPCK1 was knocked down, and then Nur77 expression levels were analyzed by western blot in Huh7 and SMMC-7721 cells.

(k) Demonstrations of stable expressing PEPCK1 in SMMC-7721 and Huh7 cells (left), and stable expressing Nur77 in PEPCK1 knockdown Huh7 cells (right). PEPCK1 and Nur77 expression levels were determined by western blot.

(l)-(m) Glucose uptake and production, lactate excretion, ECAR, OCR and ATP level were separately measured in Huh7 cells with PEPCK1 overexpression (top) or knockdown (bottom) (l). The seahorse tracing curves were shown (m).

(n) PEPCK1 inhibits proliferation of HCC cells. PEPCK1 was stably overexpressed in SMMC-7721 cells, and cell proliferation was determined by colony formation (left) and cell counting (right).

(o) The seahorse tracing curves for ECAR and OCR in Huh7 cells with PEPCK1 knockdown or/and Nur77 overexpression.

(p) PEPCK1 was knocked down first and then Nur77 2G was overexpressed in Huh7 cells. The cell proliferation was determined by colony formation.

Tubulin was used to indicate the amount of loading proteins. Data were represented as means

± SEM of at least three independent experiments. *: $p < 0.05$; **: $p < 0.01$; ***: $p < 0.001$. The data were analyzed using two-tailed Student's t-test in **b, c, l,** and one-way ANOVA followed by Tukey post hoc test in **a, e, m-o**.

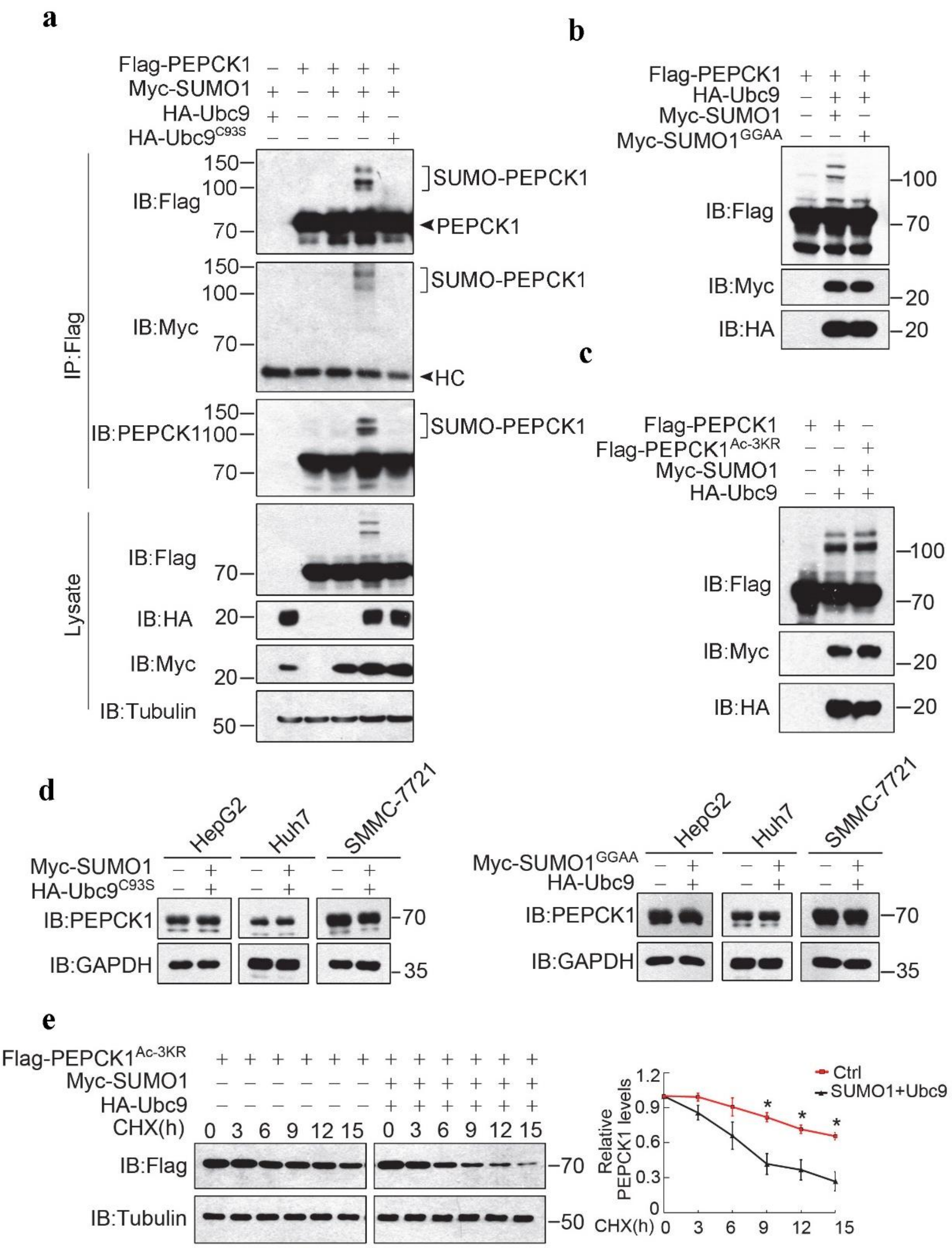
a
Flag-PEPCK1
Myc-SUMO1
HA-Ubc9
HA-Ubc9C93S
IP:Flag
IB:Flag
SUMO-PEPCK1
PEPCK1
IB:Myc
HC
IB:PEPCK1
Lysate
IB:HA
IB:Tubulin
150
100
70
50
20
b
Myc-SUMO1GGAA
c
Flag-PEPCK1Ac-3KR
d
HepG2
Huh7
SMMC-7721
IB:GAPDH
35
e
CHX(h)
0 3 6 9 12 15
Relative PEPCK1 levels
Ctrl
SUMO1+Ubc9
1.2
0.9
0.6
0.3
0
*

f

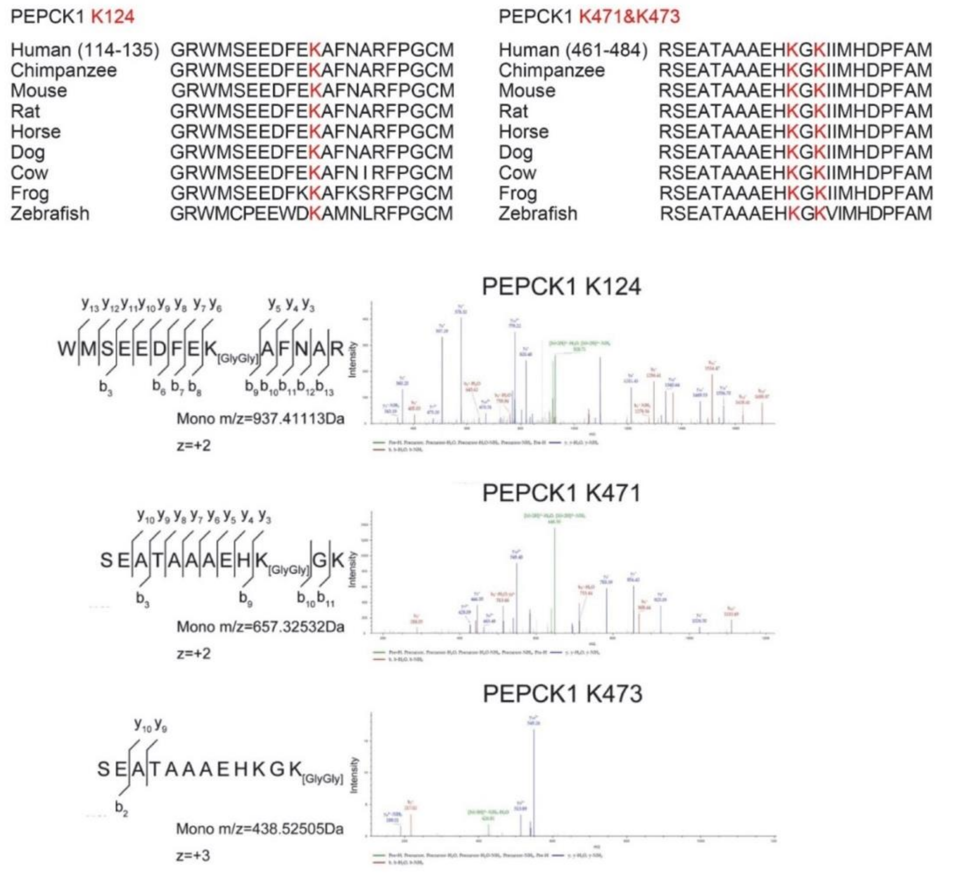

g

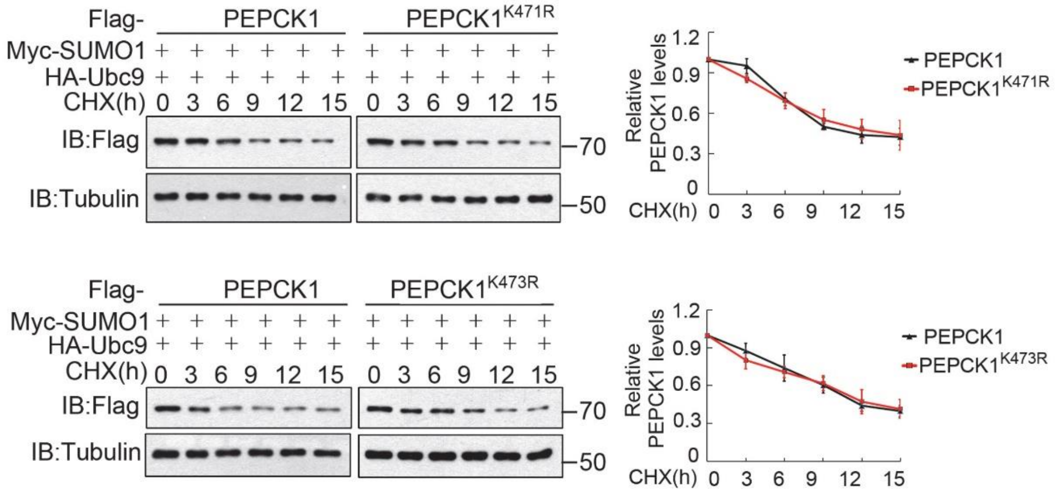

Supplementary Figure 3.

(a) SUMO1/Ubc9-induced sumoylation of exogenous PEPCK1 in 293T cells. Flag-PEPCK1 was normalized to indicate the same amount of PEPCK1 protein in each lane. Ubc9^{C93S} was used as a negative control.

(b) SUMO1^{GGAA} does not induce PEPCK1 sumoylation. PEPCK1, together with Ubc9 and SUMO1 or its mutant SUMO1^{GGAA}, was transfected into 293T cells, sumoylation of PEPCK1 was analyzed by western blot.

(c) Blocking PEPCK1 acetylation has no effect on its sumoylation. The non-acetylated PEPCK1 mutant PEPCK1$^{Ac\text{-}3KR}$, together with Ubc9 and SUMO1, was transfected into 293T cells, and sumoylation of PEPCK1 was detected by western blot.

(d) Ubc9^{C93S} or SUMO1^{GGAA} does not inhibit endogenous PEPCK1 expression. Different cell lines were transfected with SUMO1 and Ubc9 or their mutants as indicated. Expression levels of endogenous PEPCK1 were determined by western blot.

(e) Sumoylation induces PEPCK1$^{Ac\text{-}3KR}$ degradation. Different plasmids as indicated were transfected into SMMC-7721 cells, and then cells were treated with CHX (100 μg/ml) for various times. The stability of PEPCK1$^{Ac\text{-}3KR}$ was determined by western blot, and quantitated by software Image J.

(f) Identification of PEPCK1 sumoylation sites. Alignment of various PEPCK1 amino acid sequences with Lys124, Lys471 and Lys473 in red from the indicated species is shown. PEPCK1 sumoylation sites were determined by mass-spectrometric analysis. The predicted structure of SUMO1 (RGG)-conjugated PEPCK1 peptide was generated by tryptic digestion, and its b and y ions are shown in the right. The structure was confirmed by tandem mass-spectrometric analysis.

(g) Lys471 or Lys473 has no effect on PEPCK1 degradation. SMMC-7721 cells were transfected with PEPCK1 or its mutants, together with SUMO1 and Ubc9, and then treated with CHX (100 μg/ml) for indicated times. The stability of PEPCK1^{K471R} or PEPCK1^{K473R}

was determined by western blot, and quantitated by software Image J. PEPCK1 was used as a positive control.

NEM (20 mM) was added to cell lysates for repression of de-sumoylation in each sumoylation assay. Tubulin or GAPDH was used to indicate the amount of loading proteins. Data were represented as means ± SEM of at least three independent experiments. *: $p < 0.05$. The data were analyzed using one-way ANOVA followed by Tukey post hoc test.

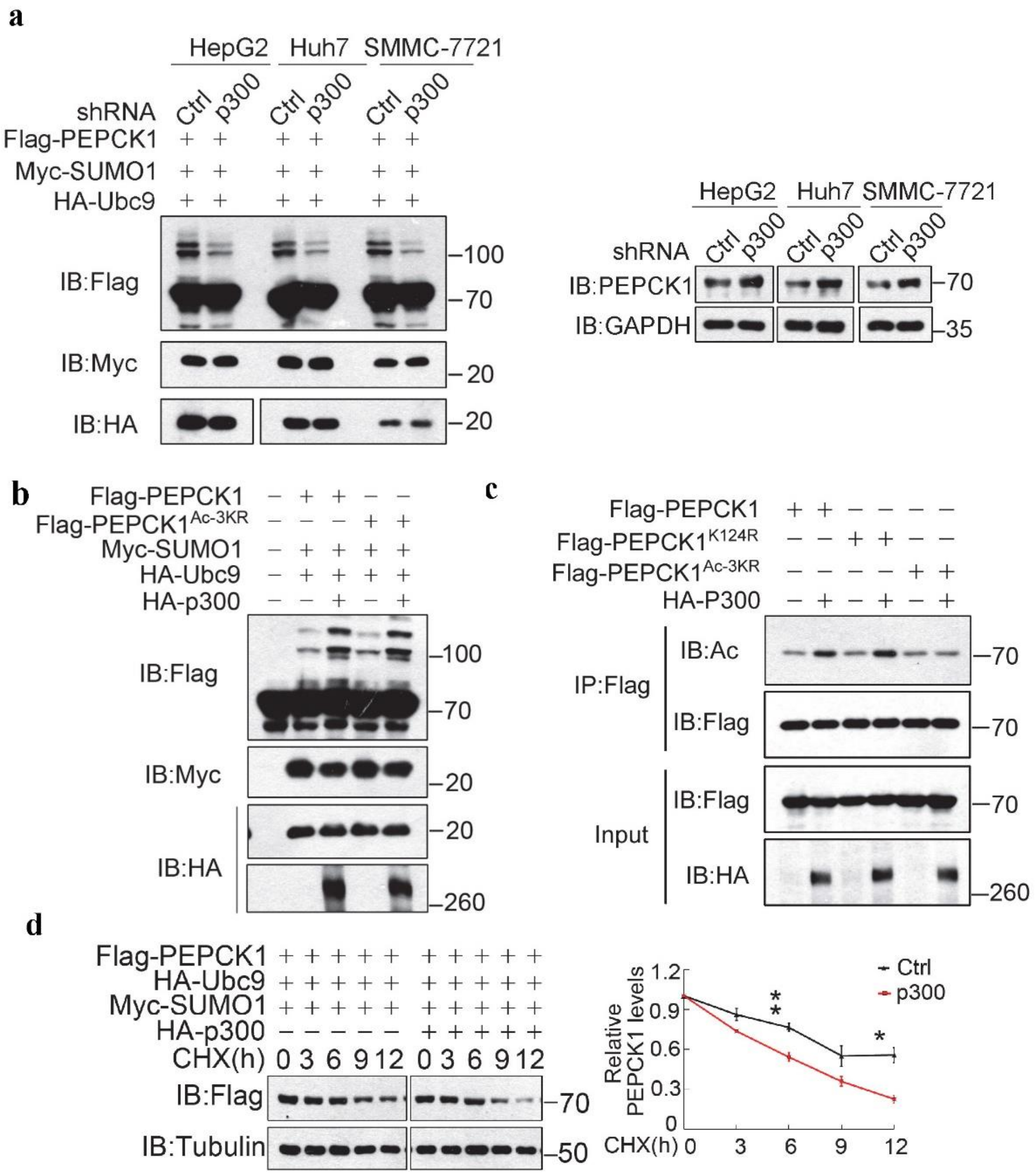
a
HepG2
Huh7
SMMC-7721
shRNA
Ctrl
p300
Flag-PEPCK1
Myc-SUMO1
HA-Ubc9
IB:Flag
IB:Myc
IB:HA
100
70
20
IB:PEPCK1
IB:GAPDH
35
b
Flag-PEPCK1
Flag-PEPCK1Ac-3KR
HA-p300
260
c
Flag-PEPCK1K124R
HA-P300
IP:Flag
IB:Ac
Input
d
CHX(h)
IB:Tubulin
50
Relative PEPCK1 levels
Ctrl
p300
0 3 6 9 12

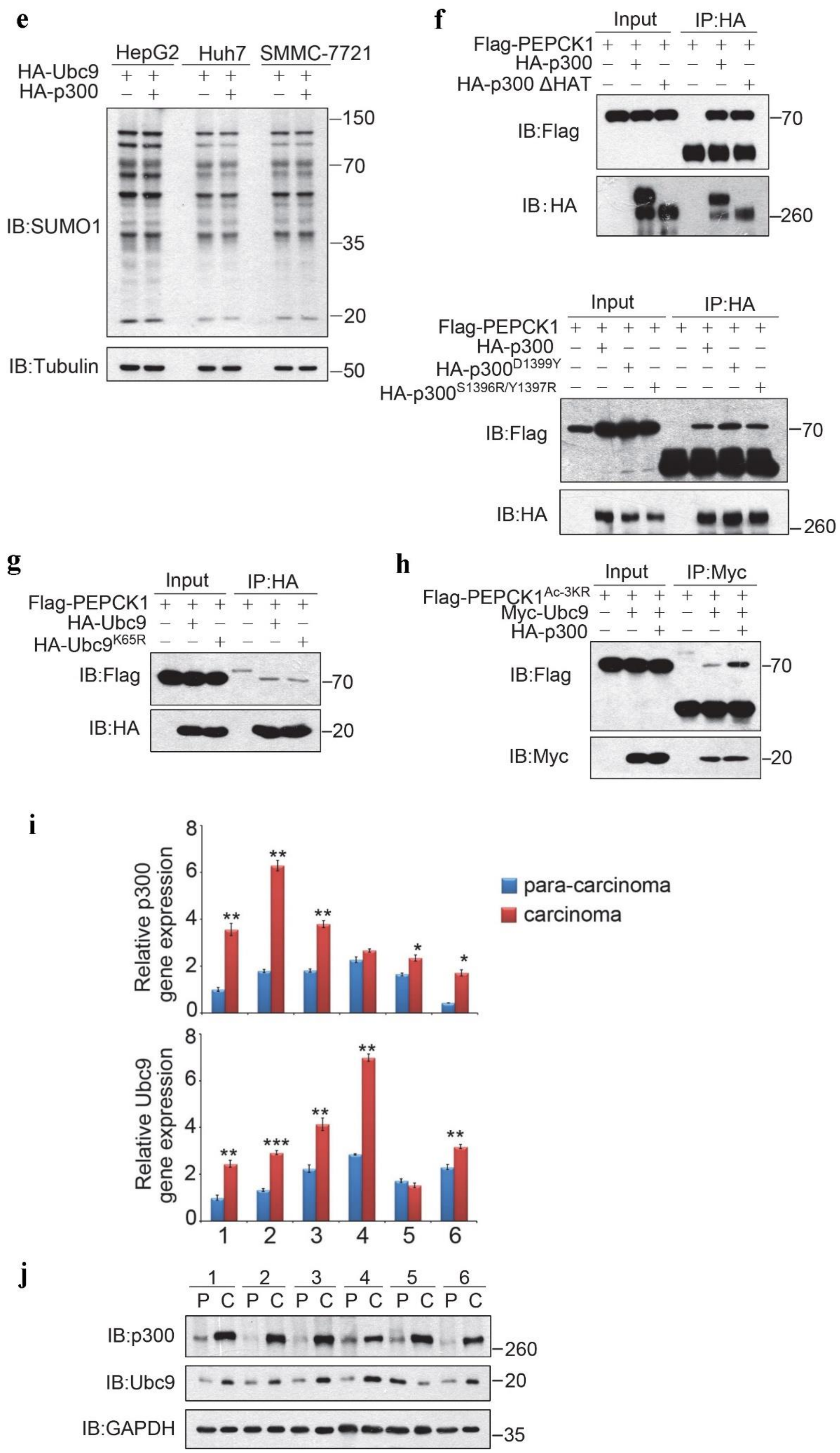
e
HepG2
Huh7
SMMC-7721
HA-Ubc9
HA-p300
IB:SUMO1
IB:Tubulin
150
70
35
20
50
f
Input
IP:HA
Flag-PEPCK1
HA-p300
HA-p300 ΔHAT
IB:Flag
IB:HA
70
260
Input
IP:HA
Flag-PEPCK1
HA-p300
HA-p300D1399Y
HA-p300S1396R/Y1397R
IB:Flag
IB:HA
70
260
g
Input
IP:HA
Flag-PEPCK1
HA-Ubc9
HA-Ubc9K65R
IB:Flag
IB:HA
70
20
h
Input
IP:Myc
Flag-PEPCK1Ac-3KR
Myc-Ubc9
HA-p300
IB:Flag
IB:Myc
70
20
i
Relative p300 gene expression
Relative Ubc9 gene expression
para-carcinoma
carcinoma
0
2
4
6
8
1
2
3
4
5
6
j
1
2
3
4
5
6
P C P C P C P C P C P C
IB:p300
IB:Ubc9
IB:GAPDH
260
20
35

Supplementary Figure 4.

(a) p300 effects on PEPCK1 sumoylation and expression in several HCC cells. p300 was knocked down in HCC cells, and the sumoylation of PEPCK1 (left) and endogenous PEPCK1 protein level (right) were shown by western blot.

(b) p300 enhances sumoylation of the non-acetylated PEPCK1 mutant. PEPCK1$^{Ac\text{-}3KR}$, together with Ubc9, SUMO1 and p300, was transfected into 293T cells, and sumoylation was determined by western blot. PEPCK1 was used as a positive control.

(c) Mutation of PEPCK1 at Lys124 does not affect p300-induced PEPCK1 acetylation. PEPCK1^{K124R} and p300 were cotransfected into 293T cells, and then PEPCK1 were immunoprecipitated. Acetylation was detected by western blot with anti-acetylation antibody (Ac). PEPCK1 was used as a positive control, and PEPCK1$^{Ac\text{-}3KR}$ was used as a negative control.

(d) p300 attenuation on PEPCK1 stability in SMMC-7721 cells under treatment with CHX (100 μg/ml). The amount of PEPCK1 protein was quantitated by software Image J.

(e) p300 did not influence the global sumoylation level in several HCC cells. HCC cells were transfected with HA-Ubc9 with or without p300. The global sumoylation levels were determined by anti-SUMO1 antibody.

(f) p300 activity is not required for interaction with PEPCK1. PEPCK1 and p300 or its different mutants, p300 ΔHAT (top) and p300^{D1399Y}, p300$^{S1396R/1397R}$ (bottom), were transfected into 293T cells as indicated. Co-IP assays were performed to determine the p300 interaction with PEPCK1 or its mutants.

(g) PEPCK1 interacts with either Ubc9 or its mutant Ubc9^{K65R} that is resistant to

p300-induced acetylation. PEPCK1 and Ubc9 or its mutant $Ubc9^{K65R}$ were transfected into 293T cells as indicated. Co-IP assays were performed to determine their interaction abilities.

(j) p300 enhanced Ubc9 binding to PEPCK1 independent of PEPCK1 acetylation in 293T cells. $PEPCK1^{Ac\text{-}3KR}$, Ubc9 and p300 were transfected into 293T cells as indicated

(i)-(j) The mRNA and protein expression levels of p300 and Ubc9 in clinical HCC (C) and paired para-carcinoma (P) samples detected by real-time PCR (i) or western blot (j).

NEM (20 mM) was added to cell lysates for repression of de-sumoylation in each sumoylation assay. Tubulin or GAPDH was used to indicate the amount of loading proteins. Data were represented as means ± SEM of at least three independent experiments. *: $p < 0.05$; **: $p < 0.01$. The data were analyzed using one-way ANOVA followed by Tukey post hoc test.

a

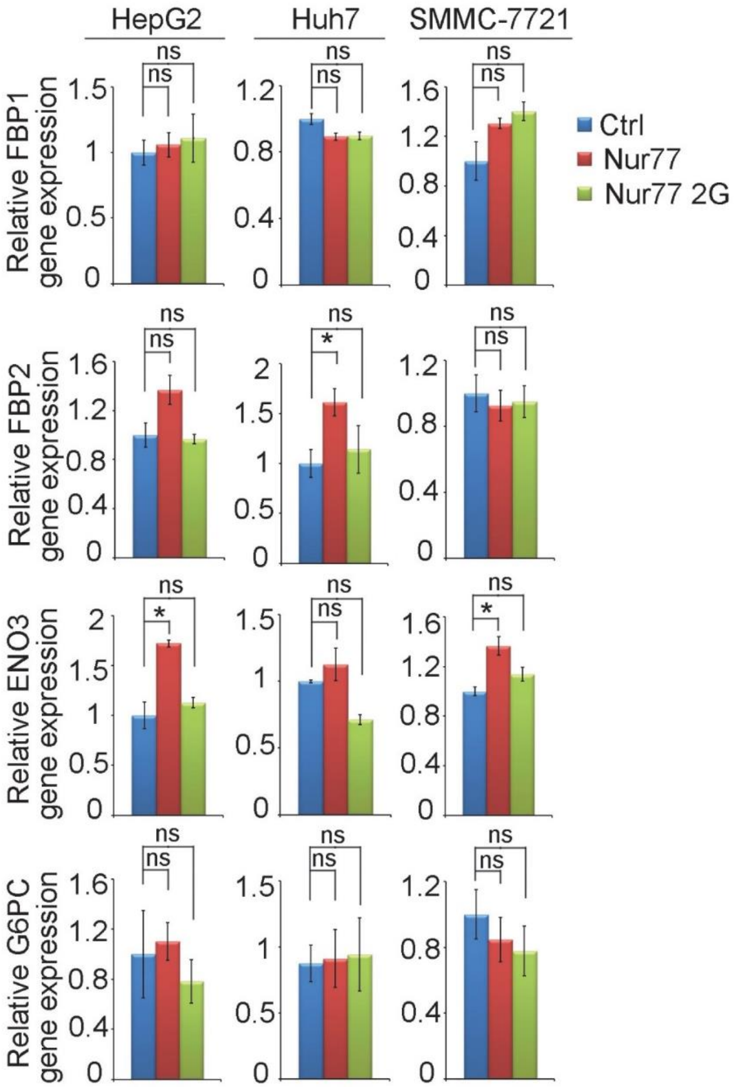
HepG2
Huh7
SMMC-7721
Ctrl
Nur77
Nur77 2G
Relative FBP1 gene expression
Relative FBP2 gene expression
Relative ENO3 gene expression
Relative G6PC gene expression
ns
*

b

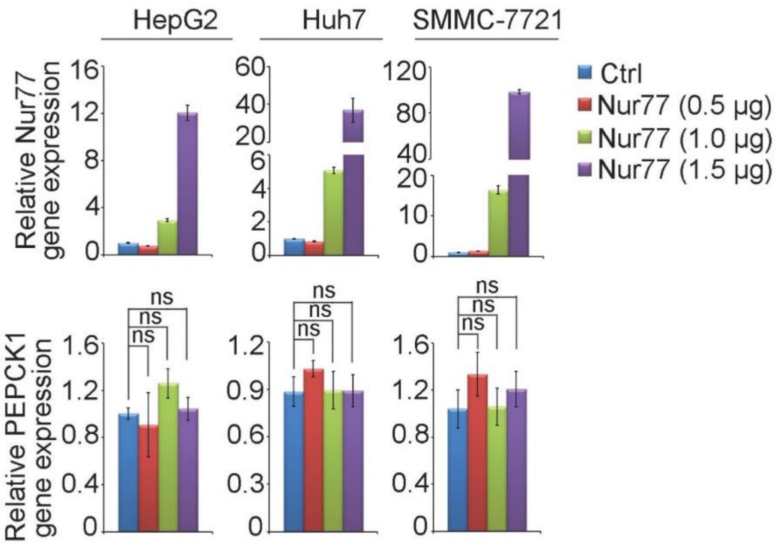
HepG2
Huh7
SMMC-7721
Ctrl
Nur77 (0.5 μg)
Nur77 (1.0 μg)
Nur77 (1.5 μg)
Relative Nur77 gene expression
Relative PEPCK1 gene expression
ns

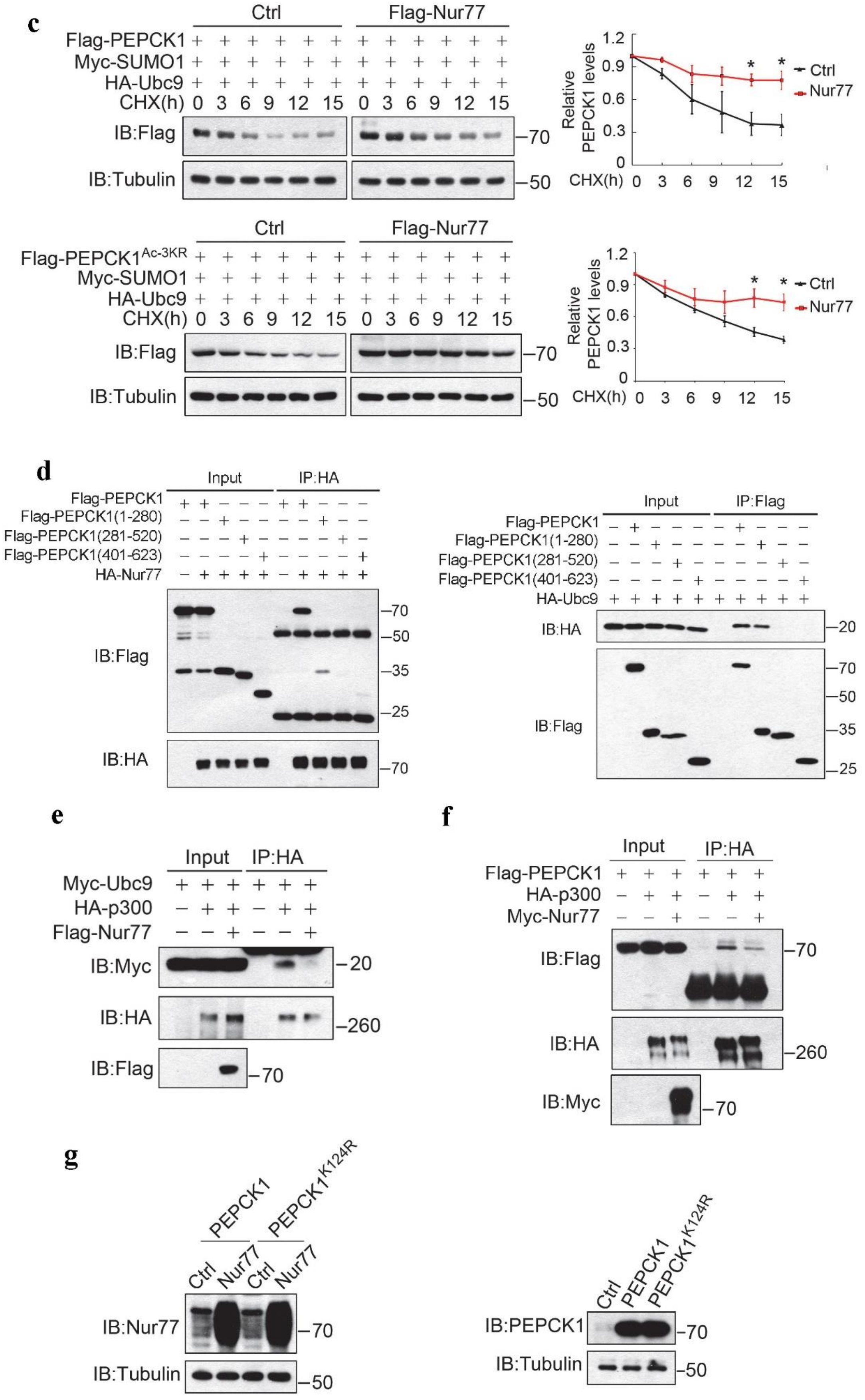
c
Ctrl
Flag-Nur77
Flag-PEPCK1
Myc-SUMO1
HA-Ubc9
CHX(h) 0 3 6 9 12 15 0 3 6 9 12 15
IB:Flag
IB:Tubulin
–70
–50
Relative PEPCK1 levels
CHX(h) 0 3 6 9 12 15
Ctrl
Nur77
Flag-PEPCK1^Ac-3KR
d
Input
IP:HA
Flag-PEPCK1
Flag-PEPCK1(1-280)
Flag-PEPCK1(281-520)
Flag-PEPCK1(401-623)
HA-Nur77
IB:Flag
IB:HA
IP:Flag
HA-Ubc9
e
Myc-Ubc9
HA-p300
Flag-Nur77
IB:Myc
IB:HA
IB:Flag
f
Myc-Nur77
g
PEPCK1
PEPCK1^K124R
Ctrl
Nur77
IB:Nur77
IB:Tubulin
IB:PEPCK1

Supplementary Figure 5.

(a) Nur77 slightly elevated mRNA levels of Fbp2 and Eno3, but not Fbp1 or G6pc in several HCC cell lines determined by realtime PCR.

(b) Overexpression of Nur77 did not influence the gene transcription levels of PEPCK1 in HCC cell lines. The mRNA levels of Nur77 (top) and PEPCK1 (bottom) were determined by realtime PCR.

(c) Transfection of Nur77 protects PEPCK1 and PEPCK1$^{Ac\text{-}3KR}$ from degradation. Nur77, together with PEPCK1 or PEPCK1$^{Ac\text{-}3KR}$, Ubc9 and SUMO1, was transfected into SMMC-7721 cells, and cells were treated with CHX (100 μg/ml) for indicated times. The stability of PEPCK1 or PEPCK1$^{Ac\text{-}3KR}$ was determined by western blot, and quantitated by software Image J.

(d) Determination of PEPCK1 interaction domains with Nur77 (left) or with Ubc9 (right). Nur77 or Ubc9, together with PEPCK1 and its deletion mutants as indicated, was transfected into 293T cells. Co-IP assays were performed to determine their interaction domains.

(e)-(f) Nur77 impairs interactions of p300-Ubc9 (e) and p300-PEPCK1 (f). Nur77, together with p300 and Ubc9 or PEPCK1 as indicated, was transfected into 293T cells. Co-IP assays were performed to determine their interaction.

(g) Stable transfection of Nur77 in PEPCK1 or PEPCK1 K124R overexpressing Huh7 cells. Nur77 and PEPCK1 expression levels were determined by western blot.

Tubulin was used to indicate the amount of loading proteins. Data were represented as means ± SEM of at least three independent experiments. *: $p < 0.05$; **: $p < 0.01$; ***: $p < 0.001$. The data were analyzed using one-way ANOVA followed by Tukey post hoc test.

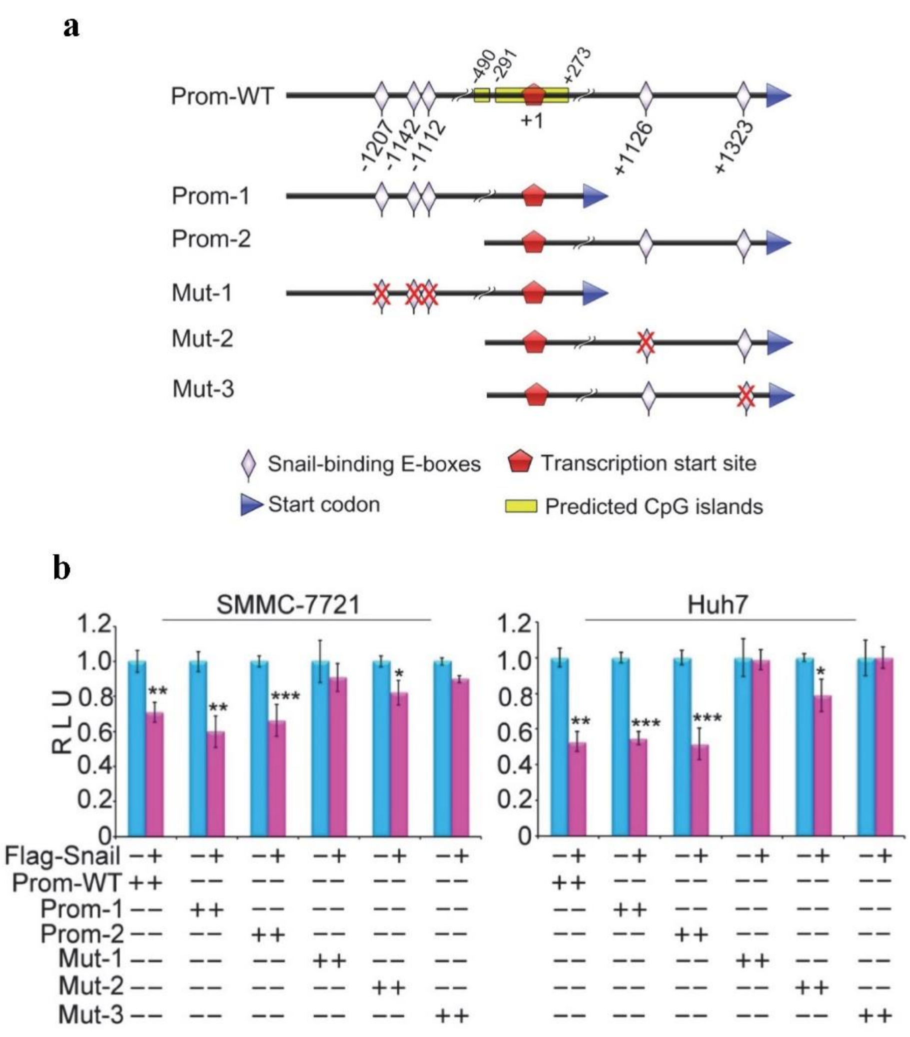

Supplementary Figure 6.

(a) Schematic diagram shows the positions of potential Snail-binding E-boxes, start codon, transcription start site, predicted CpG islands on the Nur77 promoter and different mutated constructs of luciferase reporter in the Nur77 promoter.

(b) Effects of Snail on the activity of different Nur77 promoter reporters. Cells were transfected with various plasmids, including a luciferase-linked reporter gene, a β-galactosidase (β-gal) expression vector and other vectors as indicated, and luciferase activities were measured. The β-gal activity was used to normalize for transfection efficiency.

Data were represented as means ± SEM of at least three independent experiments. *: $p < 0.05$; **: $p < 0.01$; ***: $p < 0.001$. The data were analyzed using one-way ANOVA followed by Tukey post hoc test.

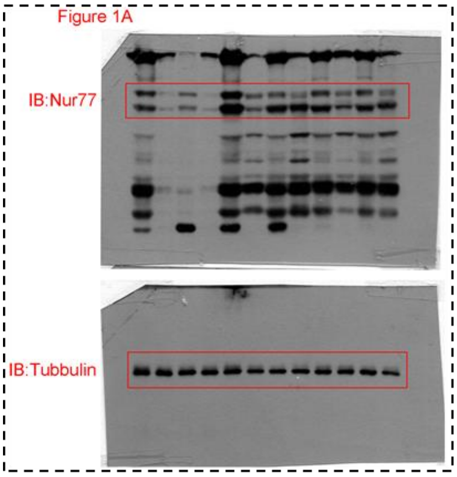
Figure 1A
IB:Nur77
IB:Tubbulin

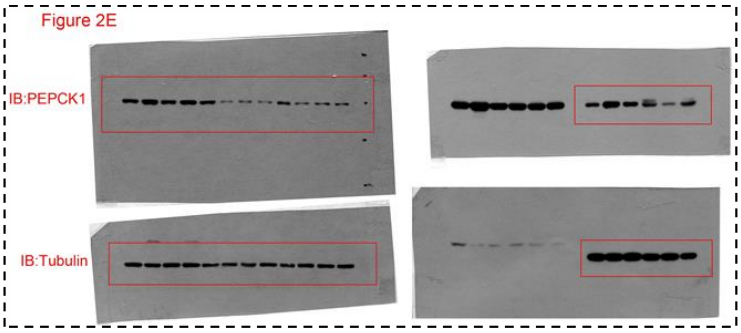
Figure 2E
IB:PEPCK1
IB:Tubulin

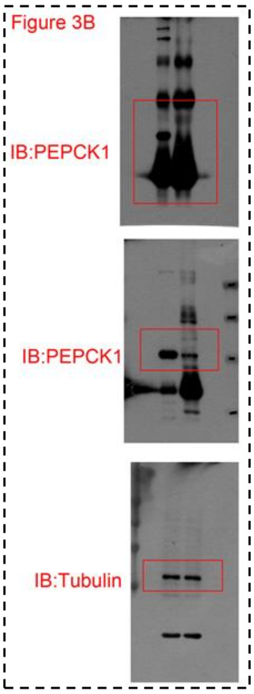
Figure 3B
IB:PEPCK1
IB:PEPCK1
IB:Tubulin

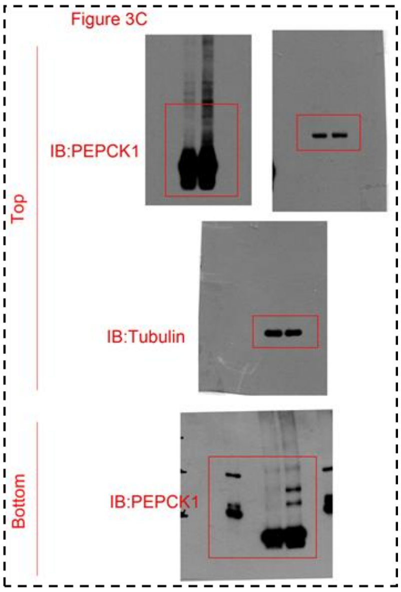
Figure 3C
Top
IB:PEPCK1
IB:Tubulin
Bottom
IB:PEPCK1

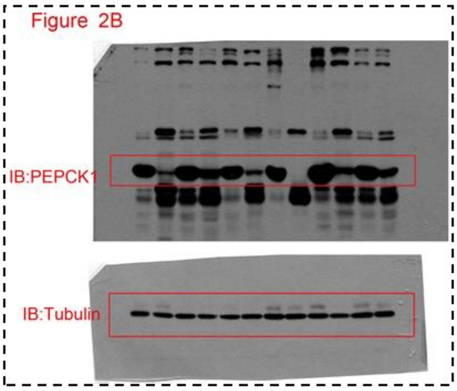
Figure 2B
IB:PEPCK1
IB:Tubulin

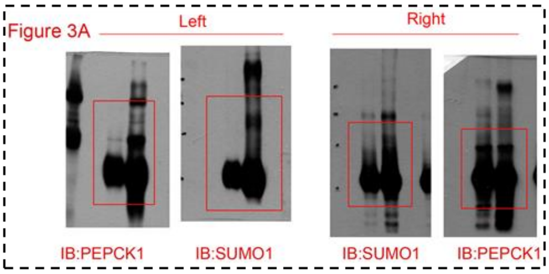
Figure 3A
Left
Right
IB:PEPCK1
IB:SUMO1
IB:SUMO1
IB:PEPCK1

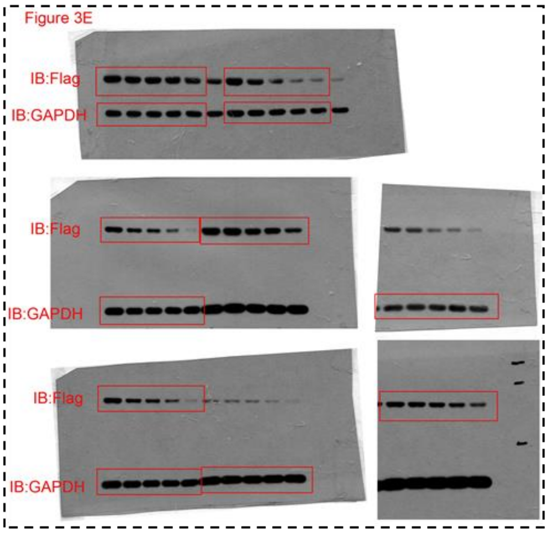
Figure 3E
IB:Flag
IB:GAPDH
IB:Flag
IB:GAPDH
IB:Flag
IB:GAPDH

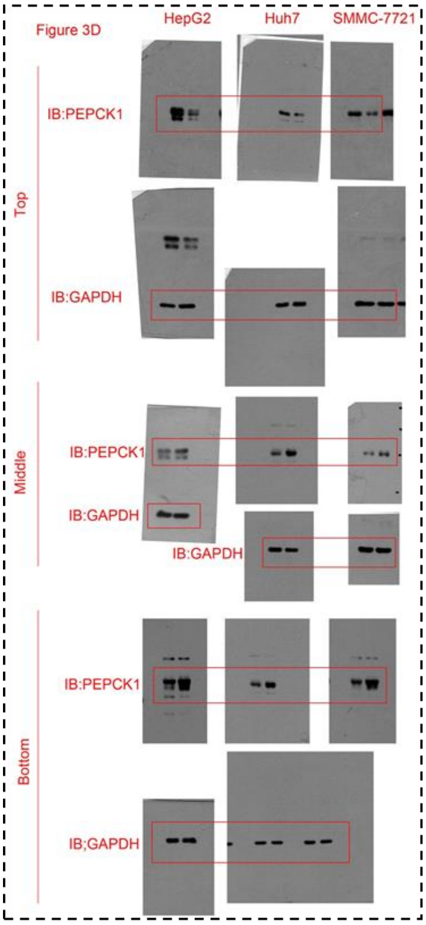
Figure 3D
HepG2
Huh7
SMMC-7721
Top
IB:PEPCK1
IB:GAPDH
Middle
IB:PEPCK1
IB:GAPDH
IB:GAPDH
Bottom
IB:PEPCK1
IB;GAPDH

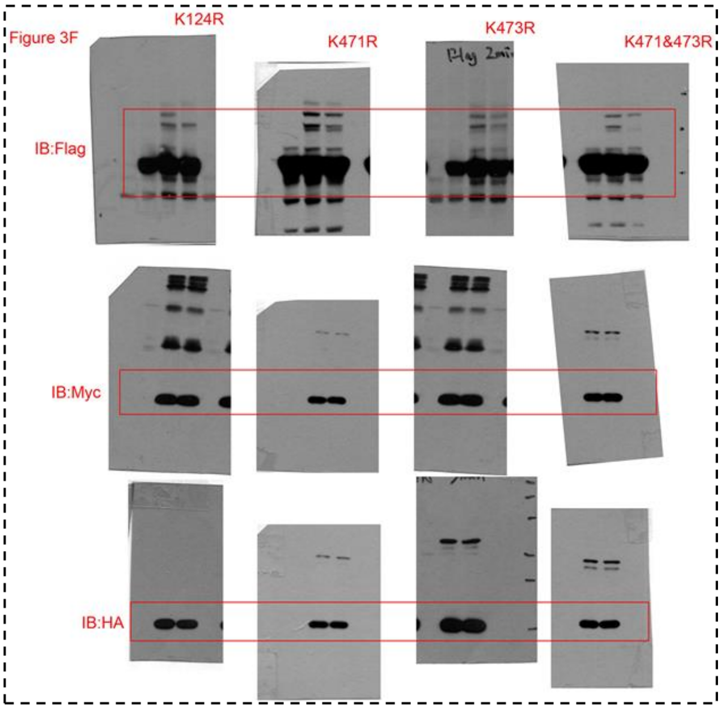
Figure 3F
K124R
K471R
K473R
K471&473R
IB:Flag
IB:Myc
IB:HA

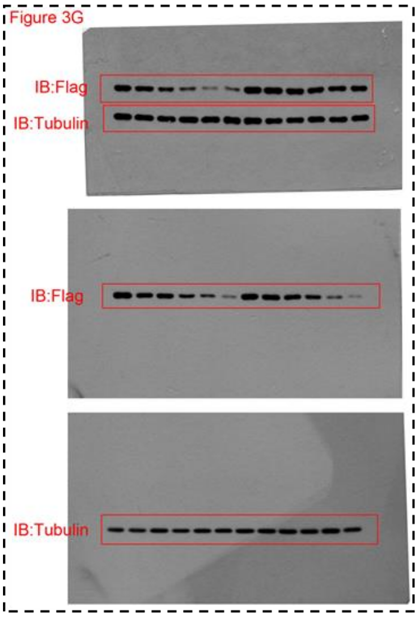
Figure 3G
IB:Flag
IB:Tubulin
IB:Flag
IB:Tubulin

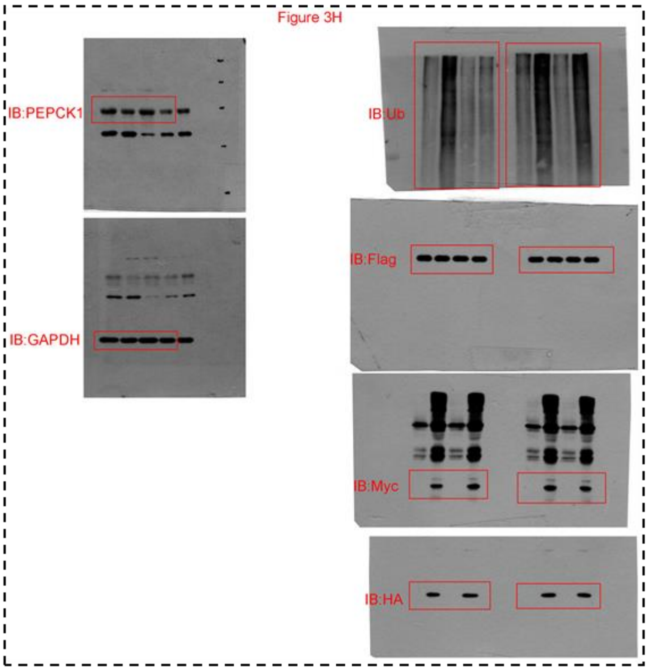
Figure 3H
IB:PEPCK1
IB:GAPDH
IB:Ub
IB:Flag
IB:Myc
IB:HA

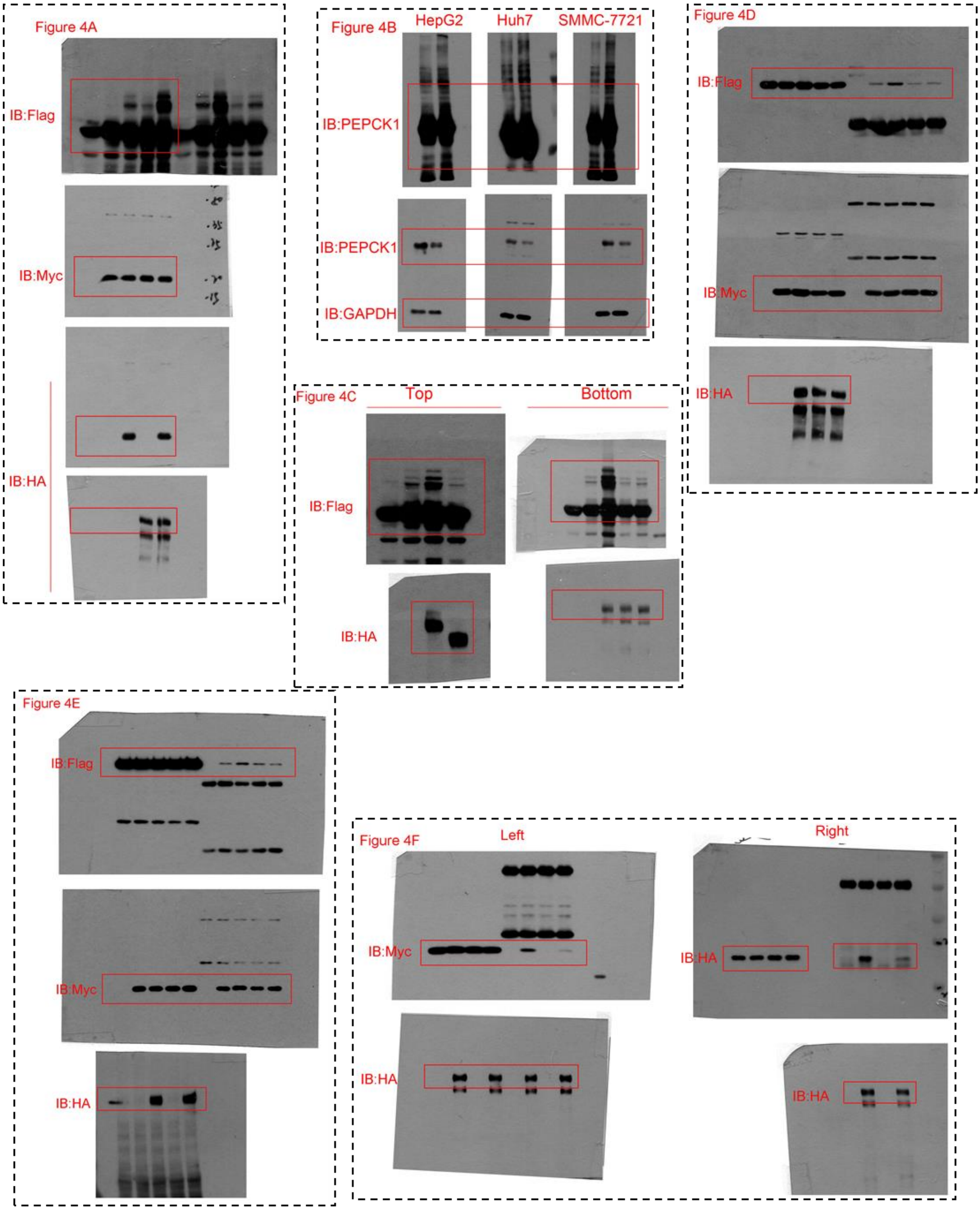
Figure 4A
IB:Flag
IB:Myc
IB:HA
Figure 4B
HepG2
Huh7
SMMC-7721
IB:PEPCK1
IB:PEPCK1
IB:GAPDH
Figure 4C
Top
Bottom
IB:Flag
IB:HA
Figure 4D
IB:Flag
IB:Myc
IB:HA
Figure 4E
IB:Flag
IB:Myc
IB:HA
Figure 4F
Left
Right
IB:Myc
IB:HA
IB:HA
IB:HA

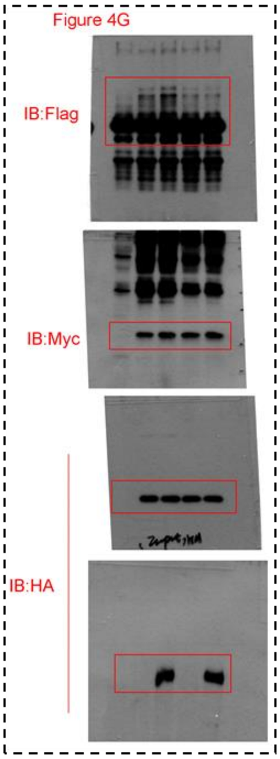
Figure 4G
IB:Flag
IB:Myc
IB:HA

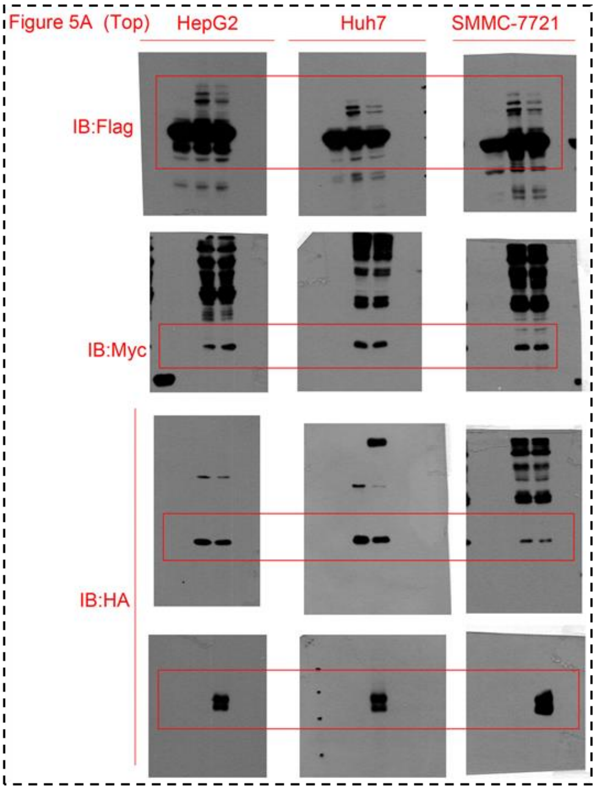
Figure 5A (Top)
HepG2
Huh7
SMMC-7721
IB:Flag
IB:Myc
IB:HA

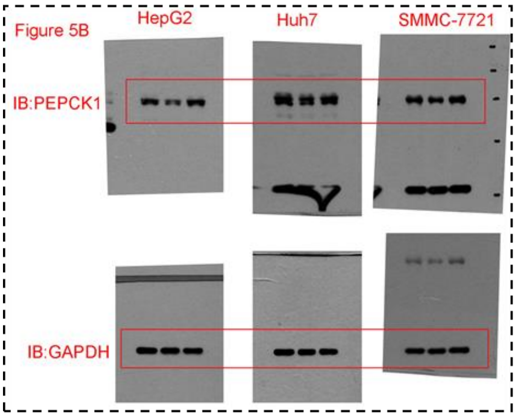
Figure 5B
HepG2
Huh7
SMMC-7721
IB:PEPCK1
IB:GAPDH

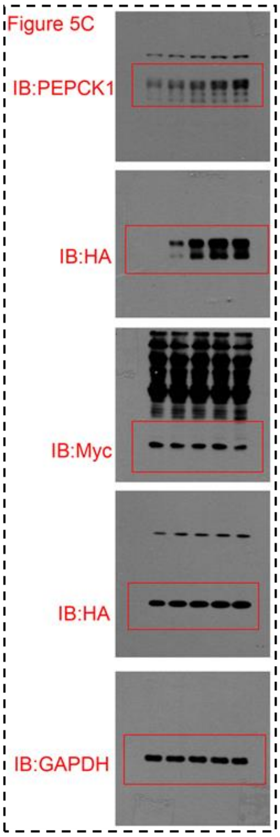
Figure 5C
IB:PEPCK1
IB:HA
IB:Myc
IB:HA
IB:GAPDH

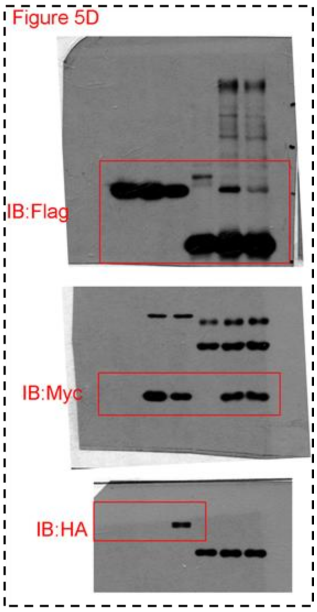
Figure 5D
IB:Flag
IB:Myc
IB:HA

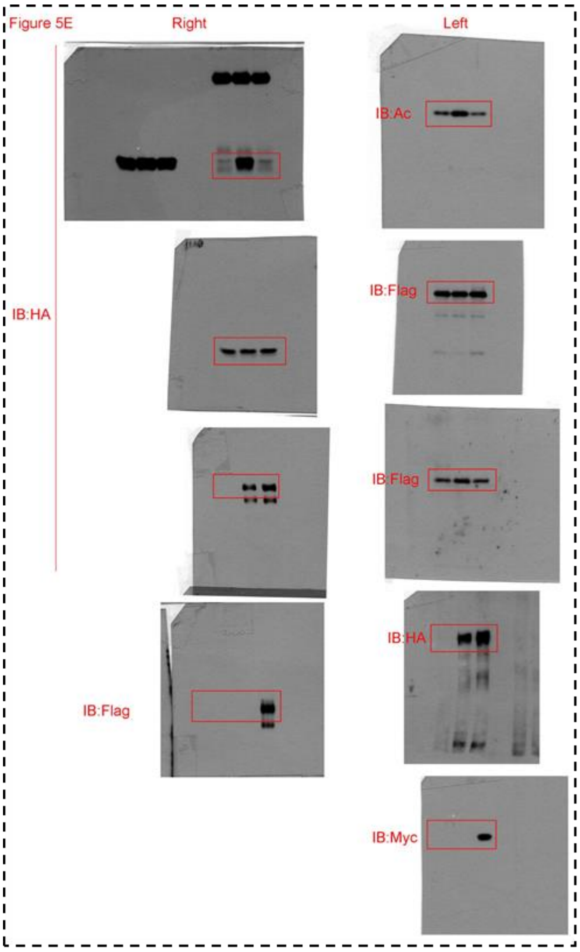
Figure 5E
Right
Left
IB:HA
IB:Ac
IB:Flag
IB:Flag
IB:HA
IB:Flag
IB:Myc

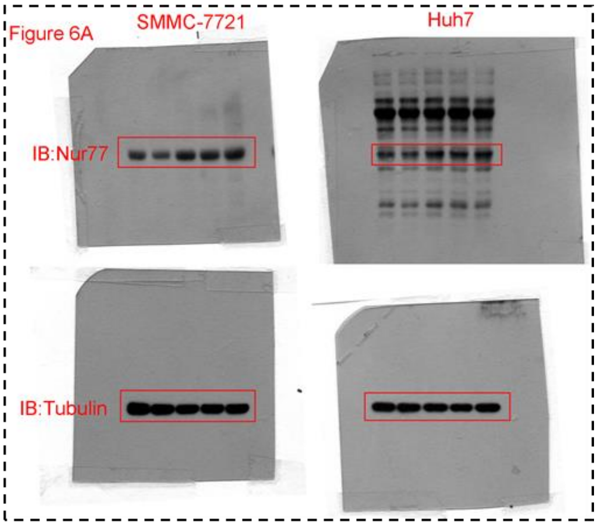
Figure 6A
SMMC-7721
Huh7
IB:Nur77
IB:Tubulin

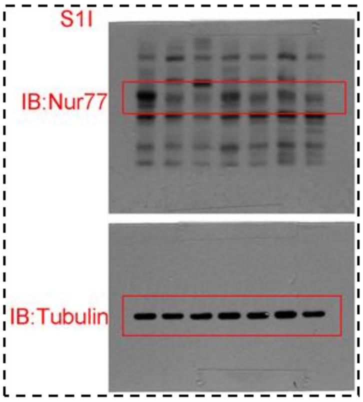
S1I
IB:Nur77
IB:Tubulin

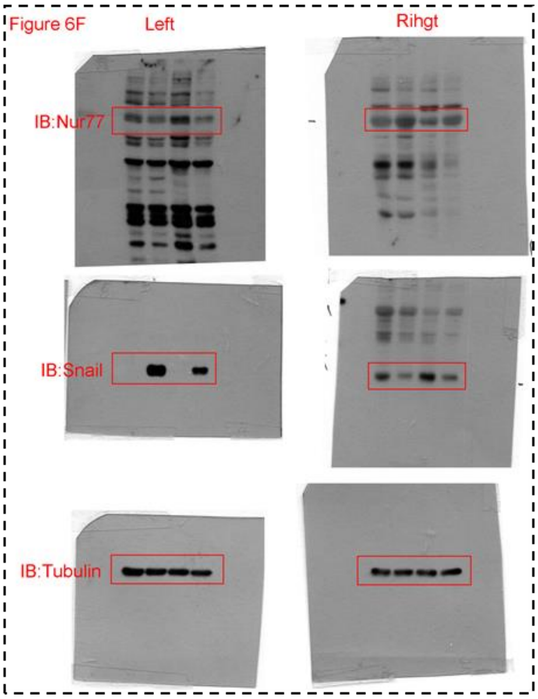
Figure 6F
Left
Rihgt
IB:Nur77
IB:Snail
IB:Tubulin

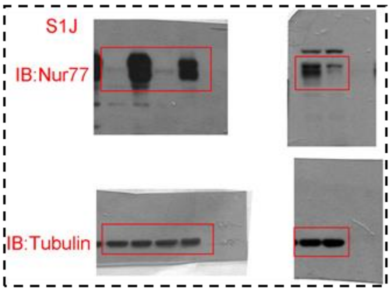
S1J
IB:Nur77
IB:Tubulin

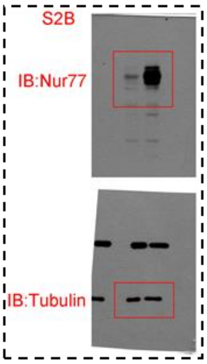
S2B
IB:Nur77
IB:Tubulin

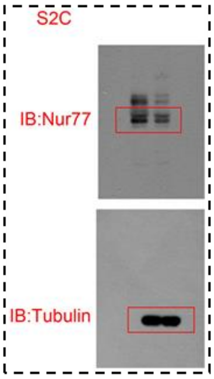
S2C
IB:Nur77
IB:Tubulin

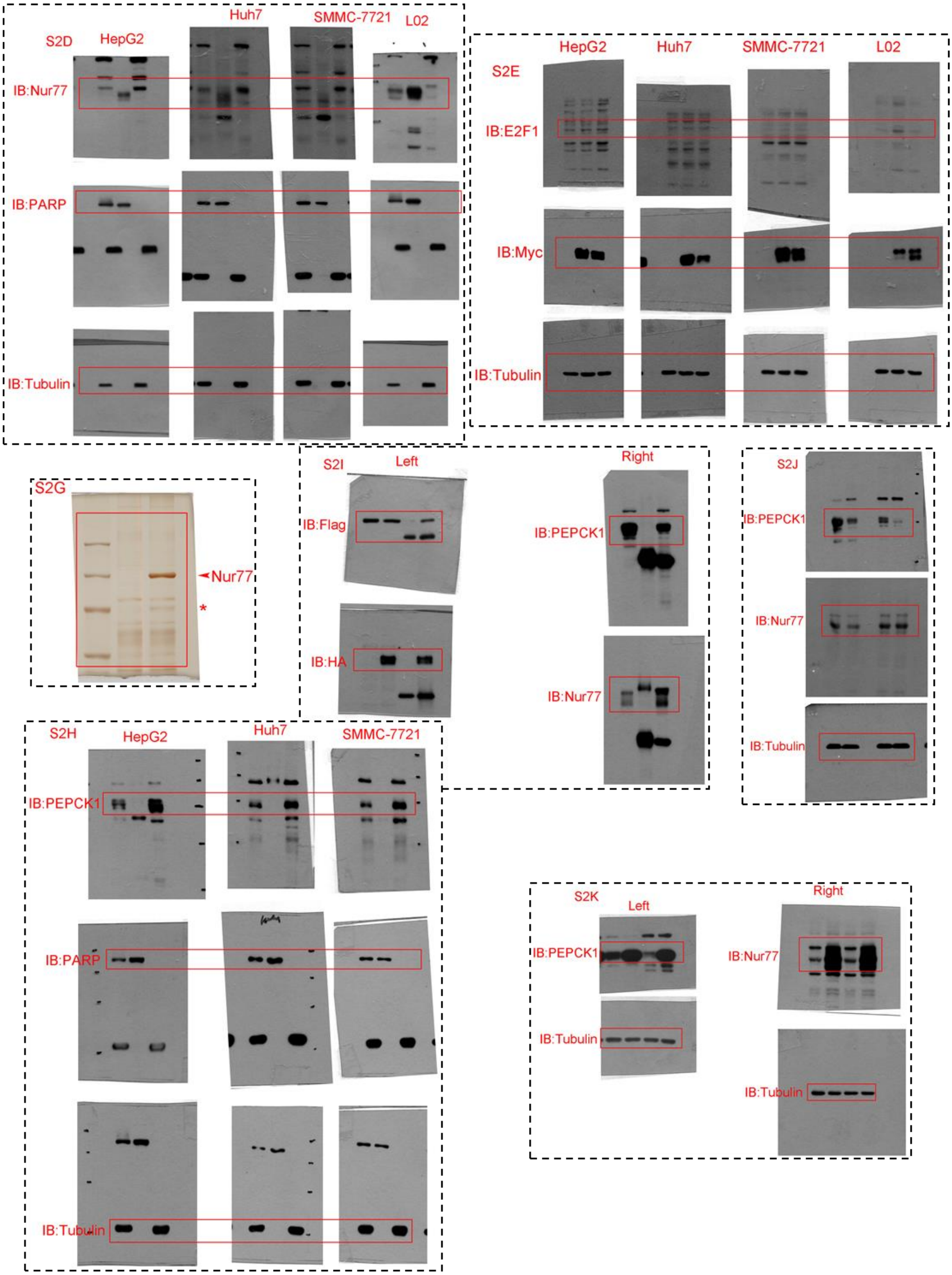
S2D
HepG2
Huh7
SMMC-7721
L02
IB:Nur77
IB:PARP
IB:Tubulin
S2E
HepG2
Huh7
SMMC-7721
L02
IB:E2F1
IB:Myc
IB:Tubulin
S2G
Nur77
*
S2I
Left
Right
IB:Flag
IB:HA
IB:PEPCK1
IB:Nur77
S2J
IB:PEPCK1
IB:Nur77
IB:Tubulin
S2H
HepG2
Huh7
SMMC-7721
IB:PEPCK1
IB:PARP
IB:Tubulin
S2K
Left
Right
IB:PEPCK1
IB:Tubulin
IB:Nur77
IB:Tubulin

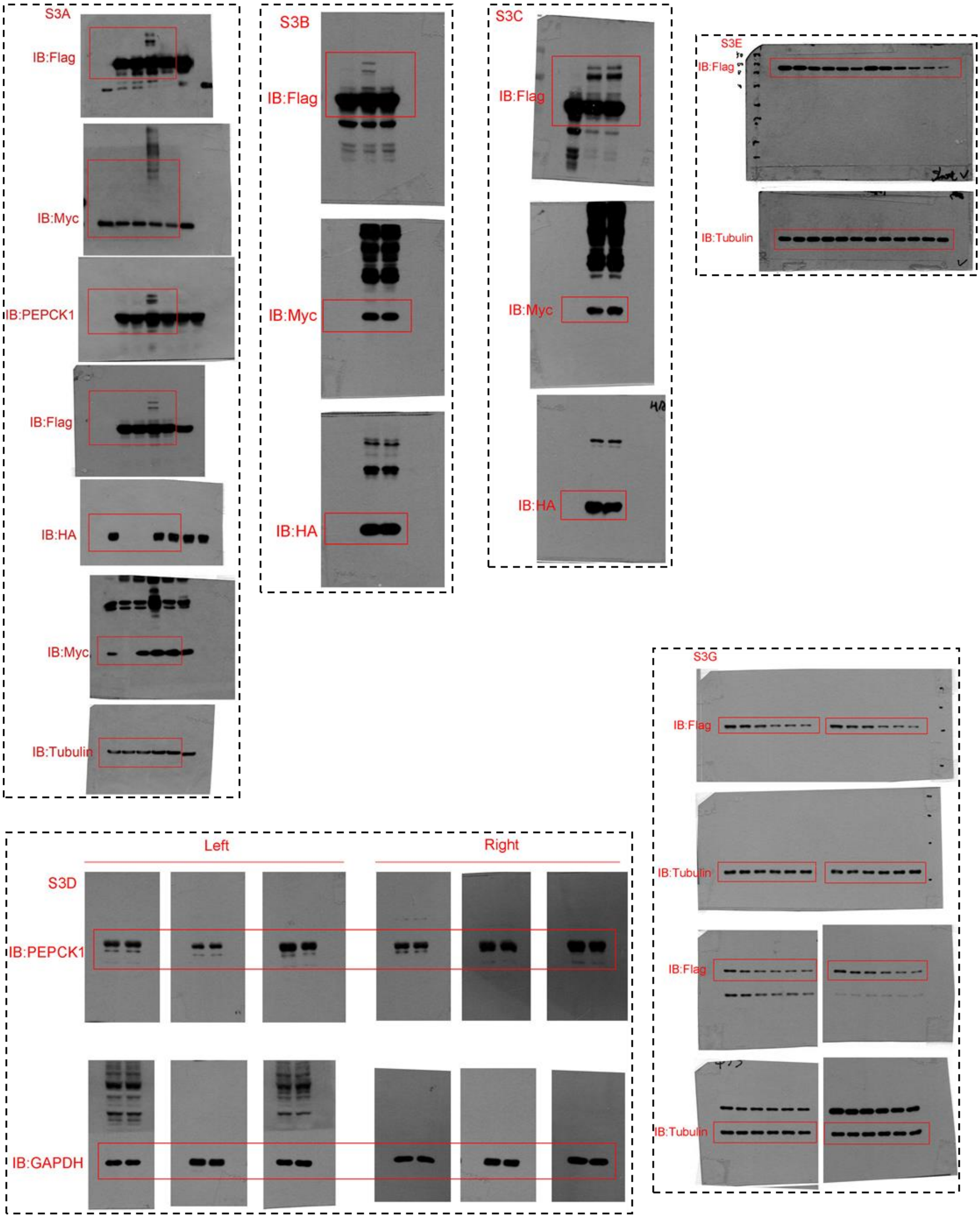
S3A
IB:Flag
IB:Myc
IB:PEPCK1
IB:Flag
IB:HA
IB:Myc
IB:Tubulin
S3B
IB:Flag
IB:Myc
IB:HA
S3C
IB:Flag
IB:Myc
IB:HA
S3E
IB:Flag
IB:Tubulin
S3D
Left
Right
IB:PEPCK1
IB:GAPDH
S3G
IB:Flag
IB:Tubulin
IB:Flag
IB:Tubulin

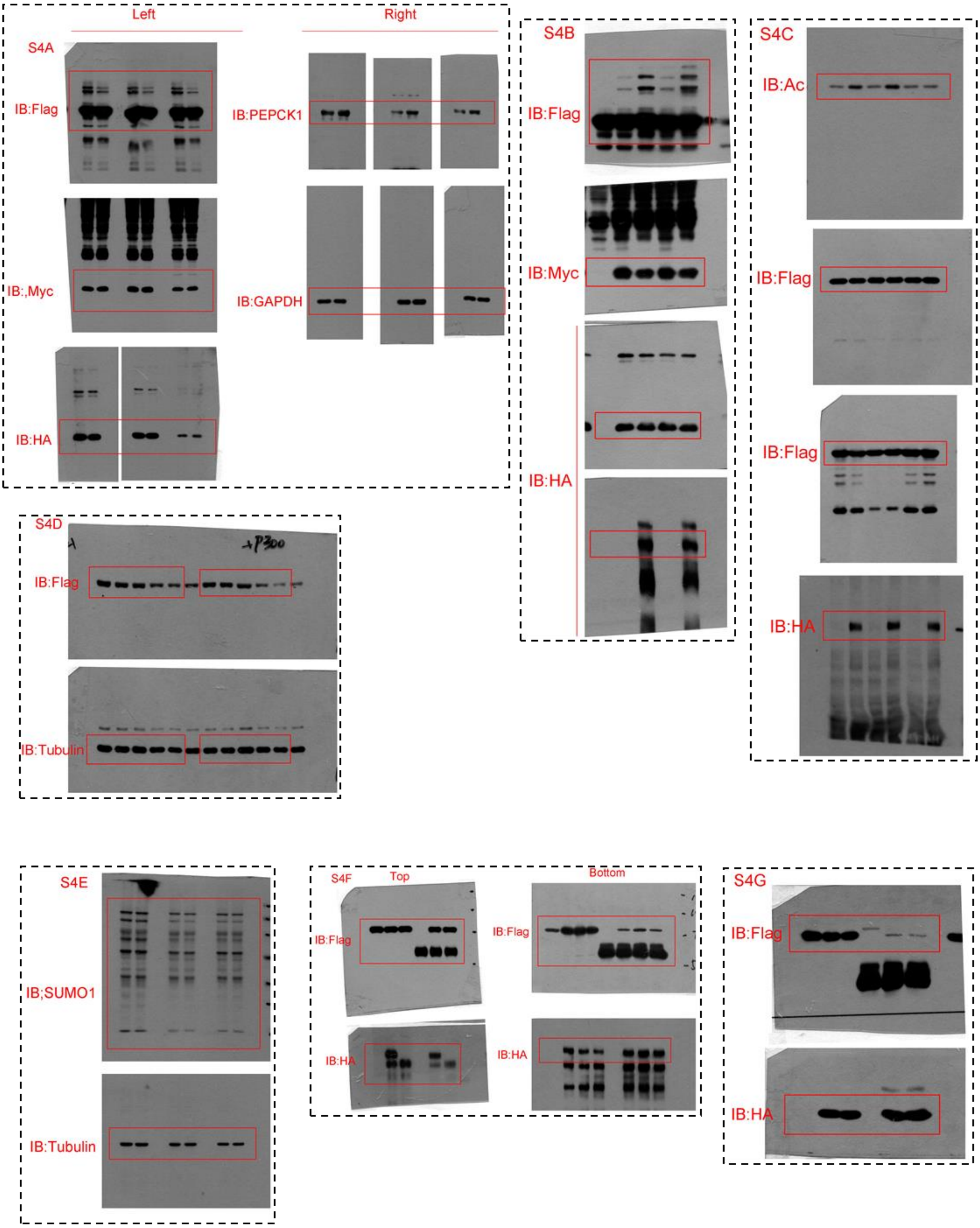
S4A
Left
Right
IB:Flag
IB:,Myc
IB:HA
IB:PEPCK1
IB:GAPDH
S4B
IB:Flag
IB:Myc
IB:HA
S4C
IB:Ac
IB:Flag
IB:Flag
IB:HA
S4D
+P300
IB:Flag
IB:Tubulin
S4E
IB;SUMO1
IB:Tubulin
S4F
Top
Bottom
IB:Flag
IB:HA
IB:Flag
IB:HA
S4G
IB:Flag
IB:HA

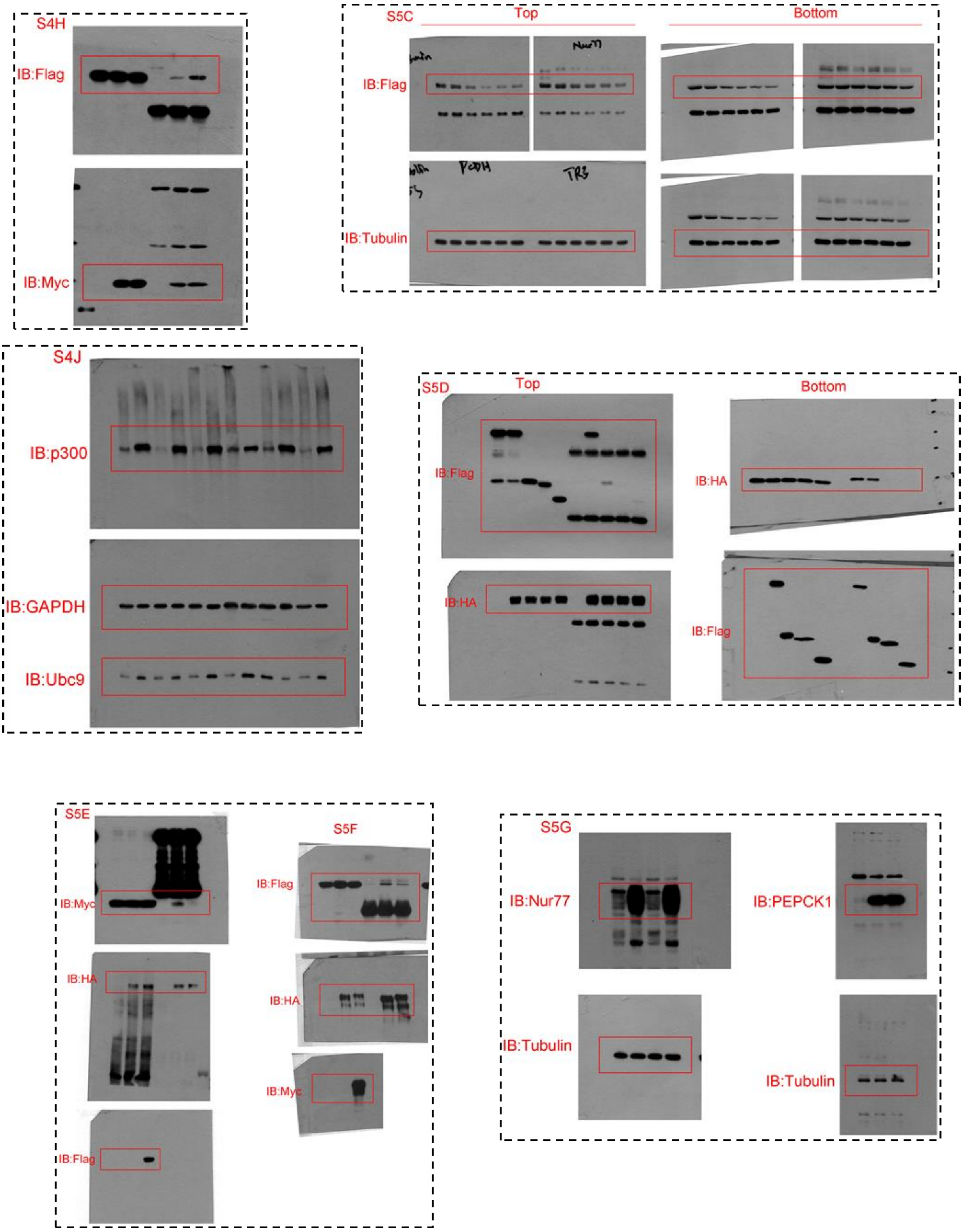

Supplementary Figure 7.

Supplementary Table 1. Correlation between Nur77 expression and information of patients with HCC.

Status		Nur77 expression#,n			Total	P value*
		NEG	MIL	M&S	n	
Sex	Male	29	65	42	136	0.2772
	Female	4	8	11	23	
Age	<55 yrs.	22	36	24	82	0.1354
	≥55 yrs.	11	37	29	77	
Tumor Size (diameter)	<6 cm	12	31	36	79	0.0043
	≥6 cm	21	42	17	80	
Survival	≤1 yrs.	6	26	5	37	0.0010
	1-3 yrs.	20	26	22	68	
	>3 yrs.	7	21	26	54	

*P values were analyzed by Chi-square test.

#According to the immunoreactive scores (IRS) from IHC of clinical samples: NEG, negative (0-1); MIL, mild (2-3); M&S, moderate (4-8) and strong (9-12).

Supplementary Table 2. Subcellular distribution of Snail in HCC and para-cancerous liver tissue from paired clinical samples.

Snail expression	Tumor		Normal		P value
	n	%	n	%	
N>C	30	36.6	24	29.3	0.0061
N<C	19	23.2	38	46.3	
N≈C	33	40.2	20	24.4	
Total	82	100	82	100	

Relative Snail expression in nucleus (N) and cytoplasm(C).
P value were analyzed by Chi-square test.
